# Nano-crumples induced Sn-Bi bimetallic interface pattern with moderate electron bank for highly efficient CO_2_ electroreduction

**DOI:** 10.1038/s41467-022-29861-w

**Published:** 2022-05-05

**Authors:** Bohua Ren, Guobin Wen, Rui Gao, Dan Luo, Zhen Zhang, Weibin Qiu, Qianyi Ma, Xin Wang, Yi Cui, Luis Ricardez–Sandoval, Aiping Yu, Zhongwei Chen

**Affiliations:** 1 Guangdong Provincial Key Laboratory of Nanophotonic Functional Materials and Devices, School of Information and Optoelectronic Science and Engineering & International Academy of Optoelectronics at Zhaoqing, South China Normal University, 510631 Guangdong, China; 2 Department of Chemical Engineering, Waterloo Institute for Nanotechnology, Waterloo Institute for Sustainable Energy, University of Waterloo, 200 University Avenue West, Waterloo, ON N2L 3G1 Canada; 3 South China Academy of Advanced Optoelectronics, South China Normal University, 510006 Guangdong, China; 4 Vacuum Interconnected Nanotech Workstation, Suzhou Institute of Nano-Tech and Nano-Bionics, Chinese Academy of Sciences, 215123 Suzhou, China

**Keywords:** Electrocatalysis, Density functional theory, Electrocatalysis

## Abstract

CO_2_ electroreduction reaction offers an attractive approach to global carbon neutrality. Industrial CO_2_ electrolysis towards formate requires stepped-up current densities, which is limited by the difficulty of precisely reconciling the competing intermediates (COOH* and HCOO*). Herein, nano-crumples induced Sn-Bi bimetallic interface-rich materials are in situ designed by tailored electrodeposition under CO_2_ electrolysis conditions, significantly expediting formate production. Compared with Sn-Bi bulk alloy and pure Sn, this Sn-Bi interface pattern delivers optimum upshift of Sn p-band center, accordingly the moderate valence electron depletion, which leads to weakened Sn-C hybridization of competing COOH* and suitable Sn-O hybridization of HCOO*. Superior partial current density up to 140 mA/cm^2^ for formate is achieved. High Faradaic efficiency (>90%) is maintained at a wide potential window with a durability of 160 h. In this work, we elevate the interface design of highly active and stable materials for efficient CO_2_ electroreduction.

## Abstract (web summary)

It is of high interests to design catalysts for CO2 electroreduction with enhanced selectivity and activity. Here, the authors report Sn-Bi bimetallic interface-rich material with enhanced performance for CO2 reduction to formate comparing to that of Sn-Bi bulk alloy.

## Introduction

The development of carbon dioxide (CO_2_) conversion strategies to produce low carbon chemicals and fuels is one of the most important solutions considered to effectively alleviate worldwide carbon emissions^1–4^. CO_2_ electroreduction reaction (CO_2_RR) is an emerging sustainable technology that can be utilized not only to upgrade CO_2_ but also to store intermittent electricity produced by renewable energy^5–7^. Among the variety of possible products, formic acid or formate is of great interest owing to its wide applications in commercialized synthesis industries and formic acid fuel cells, with relatively low activation potentials needed (only 2-electron transfer)^8,9^. The binary metallic catalysts offer an effective approach to tune the selectivity and activity of CO_2_-to-formate transformation^10,11^, e.g., Pd–Sn alloy^12^, Pd–Pt bimetallic nanoparticles^13^, Ag–Sn core–shell structures^8^, etc. However, the industrial application of this process relies on inexpensive catalysts, stepped-up partial current density, and prolonged stability.

Our previous work reported low-cost Sn nanosheets decorated with Bi nanoparticles achieving a high Faradaic efficiency towards formate (FE_formate_) of 96% at −1.1 V versus reversible hydrogen electrode (RHE)^14^. Hitherto, it still remains challenging to delicately regulate the adsorption of coupled competing intermediates (COOH and HCOO), which is critical to further enhance the current density at the low potential for nanostructured Sn–Bi materials. As reported, the catalytic properties are highly related to the atomic patterns, because the intermetallic interactions occurring at the interfaces within two metals are fundamentally different from bulk alloys^15,16^, e.g., bimetallic Au–Cu electrocatalyst exhibits higher synergistic activity and selectivity of C_2+_ products compared to Au–Cu alloys ascribed to the stabilization of key intermediate species, *COOH towards CO formation^17–19^.

Herein, in an attempt to gain a comprehensive understanding of the correlation between the catalytic activities of Sn–Bi system with their electronic structures and bimetallic patterns, we performed ab initio calculations, and identified that Sn interacting Bi at the interface is the most favorable structure for HCOOH formation. Such structure was proved to offer weakened Sn–C hybridization of competing for COOH* intermediate and optimum Sn–O hybridization of HCOO*. Guided by the theoretical findings and with the target of exposing a more active Sn–Bi interface, nano-crumples induced Sn–Bi interface structures were experimentally designed through in situ electrodeposition under CO_2_RR conditions. This design concept of exposing the specific surfaces under a capping agent has been proposed recently in the electrochemical systems^20–22^.

Here, we show this interface design strategy demonstrates multiple merits to tackle the aforementioned challenges: (i) to the authors’ knowledge, a high partial current density of formate (up to 140 mA/cm^2^) was achieved in an H-cell; this is attributed to abundantly exposed active Sn–Bi bimetallic interface pattern originated from the nano-wrinkles/crumples under CO_2_RR conditions. (ii) highest FE_fromate_ was obtained up to 96.4% at a lower potential (−0.84 V vs. RHE) through simultaneously suppressing the binding of COOH* towards CO formation, and enhancing the binding of HCOO* towards formate formation. (iii) high Faradaic efficiency of formate (>90%) was maintained at wide potentials window (−0.74 to −1.14 V vs. RHE) with a durability of 160 h; this is ascribed to well-defined surface structures at steady crumples grown on interconnected porous carbon fabric network, which offers expedite mass transport and electronic conductivity. This manipulation of electronic structures of active sites by nano-crumples elucidates the correlations among morphology, surface structures, electronic properties, and reaction pathways, providing a rational design strategy to enhance the catalytic performance of nanostructured materials.

## Results

### Density functional theory (DFT) calculations

**Fig. 1:**
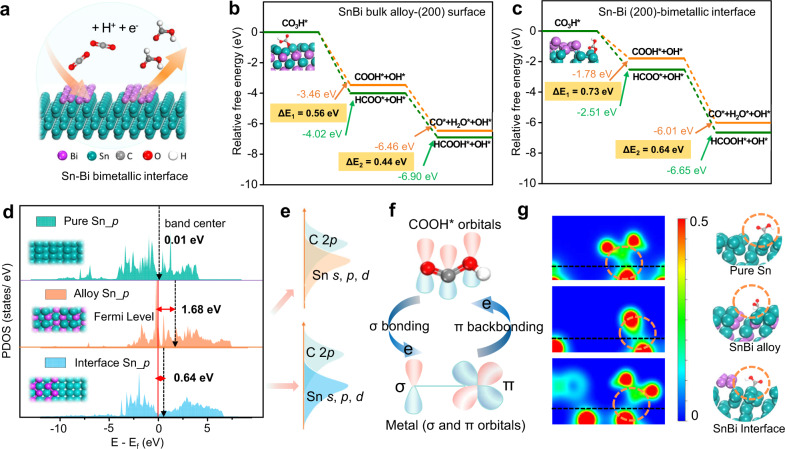
DFT simulations and proposed catalytic mechanism. **a** Scheme of CO_2_RR on Sn–Bi bimetallic interface. Gibbs free energy profiles of CO and HCOOH production pathways on (**b**) Sn–Bi alloy surface and (**c**) Sn–Bi bimetallic interface. **d** PDOS of Sn 5*p* orbitals of pure Sn, alloy Sn, and interface Sn and weighted band center for all three models without adsorbate (overall range). **e** Schematic illustration for PDOS overlapping areas of Sn *s*, *p*, and *d* orbitals on Sn–Bi alloy and Sn–Bi bimetallic interface with C 2*p* orbitals of COOH*, respectively. **f** Scheme of σ bonding and π backbonding between COOH and Sn metal. **g** Calculated volume slices of calculated charge densities and corresponding optimized configurations for the three models with COOH adsorbate. The black dashed lines indicate the positions of the metal surfaces.

The correlation between catalytic activity of the Sn–Bi binary system with the electronic properties and atomic patterns was initially evaluated using density functional theory (DFT) calculations (Fig. 1). The incorporation of Bi atoms into Sn species facilitates formate production by suppressing the formation of both H_2_ and CO^23^. Our previous work also revealed that the addition of Bi allows the HCOO* intermediate to be favorably adsorbed onto the Sn–Bi surface compared to a pure Sn surface, thus boosting HCOOH formation^14^. Nonetheless, Sn–Bi binary systems can be classified into two categories: one that exhibits the bulk-type ordered alloy crystal phase (denoted as Sn–Bi alloy) while the other is the surface alloy with the interaction only in the surface or subsurface region (referred to as Sn–Bi bimetallic interface, Fig. 1a)^16,24,25^. Thus, Sn (200) surface was built because it was reported as one of the most thermodynamically stable facets^14,23,26^. One periodic atomic layer of Bi in one direction was mounted on Sn (200) surface model to represent the interactions in Sn–Bi bimetallic interface (Supplementary Fig. 1). Sn unit cell with atomic-level uniform Bi substitution was adopted to describe the Sn–Bi bulk alloy, where the (200) alloy surface was also built to compare with Sn–Bi bimetallic interface model (Supplementary Fig. 1). We considered different combinations of Sn and Bi atoms in the unit cell and chose the one yielding the most stable Sn–Bi bulk alloy surface structure (Supplementary Table 1).

The atomic pattern effects were investigated and elaborated on the CO_2_RR electrocatalytic activities through the bimetallic Sn–Bi interface and alloy (Fig. 1b, c, and Supplementary Fig. 2). As reported^14,27^, COOH* and HCOO* are the two major competing intermediates for CO and HCOOH production, respectively. Thus, two pathways via these two intermediates are considered in this study and originated from the hydrogenation of the adsorbed bicarbonate (CO_3_H*) species, which is reported as the primary carbon source for CO_2_RR toward formate production with neutral KHCO_3_ electrolyte^12,28,29^. The free energy profiles obtained from DFT analysis demonstrate that the HCOOH formation via the HCOO* intermediate is the favorable pathway on both surfaces owing to the relatively lower free energy of this pathway. The free energy difference for the intermediates HCOO* and COOH* via the first proton-electron transfer reaction is higher on Sn–Bi bimetallic interface (0.73 eV) than on the Sn–Bi alloy surface (0.56 eV). This difference indicates that the formation of HCOO* intermediate is more thermodynamically facile compared to the formation of COOH* on the former structure. Moreover, for the second proton-electron transfer reaction step towards HCOOH* or CO* formation, the free energy difference between two competing products is also higher on Sn–Bi bimetallic interface (0.64 eV) than that on Sn–Bi alloy surface (0.44 eV). We also studied CO_2_ reduction activity on the in-plane Sn–Bi heterostructure (Supplementary Fig. 1). The results shown in that figure confirm that the Sn–Bi interface models (i.e., the current interface model and the in-plane model) all have higher activity towards formate formation than Sn–Bi bulk alloy model.

The adsorption energies (*E*_*ads*_) of three main intermediates on the two Sn–Bi surfaces and pure Sn(200) surface were calculated and listed in Supplementary Table 2. It was found that COOH* prefers bonding with the surface via C atom while CO_3_H* and HCOO* tend to bond the surface with O atom^12,28^. The adsorption of COOH is the weakest on Sn–Bi bimetallic interface (*E*_*ads*_ of −1.62 eV); the adsorption of HCOO is moderate (*E*_*ads*_ of −3.09 eV) while the adsorption of CO_3_H is the strongest (*E*_*ads*_ of −3.64 eV). To further understand these different sequences of adsorption strength for three intermediates, an electronic state analysis was conducted through the partial density of states (PDOS) for these three adsorbed systems (Supplementary Figs. 3–4). Moreover, the outermost valence *p* orbital of Sn atom on pure Sn and two Sn–Bi surface models (without adsorbates) were analyzed along with the *p* band center within the overall range of orbital^30^, as shown in Fig. 1d. It can be seen that the addition of Bi upshifts the electron states of Sn away from the Fermi level for both Sn–Bi models, suggesting the electron donation from Sn to Bi^14,31^. Notably, the p-band center of Sn–Bi interface model lies among that of pure Sn and Sn–Bi alloy, delivering a moderate oxide state or electron bank. Consequently, the PDOS of Sn–C atoms in COOH* adsorption and Sn–O atoms in HCOO* adsorption (Supplementary Fig. 3 and Fig. 1e) shows that there are more harmonic *p*-*p* and *p*-*s* overlaps between the C-2*p* and Sn-5*s*, Sn-5*p* states on Sn–Bi alloy model compared to those of Sn–Bi bimetallic interface in COOH adsorption, indicating weaker adsorption strength of COOH* of the latter^24,32^. As expected, fewer overlapping areas of *p*-*p* and *p*-*s* orbitals between O-2*p* and Sn-5*p*, Sn-5*s* for HCOO* adsorption were also found on the Sn–Bi interface compared with those of alloy.

The reason why moderate electron bank of Sn in bimetallic Sn–Bi interface results in the weakest COOH adsorption was proposed as the tailoring of metal–carbon hybridization. The electron donation from the COOH to the metal by σ bonding and π backdonation from the metal to the COOH describes the concerted coupling of the COOH levels to the metal *sp* states and the *d* states (Fig. 1f)^33–35^. Pure Sn with higher electronic density and alloy Sn in Sn–Bi with the most electronic depletion could both create stronger *p*–*d* interaction through π backbonding and σ bonding^36^, respectively, compared to Sn with moderate electronic density in Sn–Bi. This is because the sufficient electron (or hole)-donating capacity of the hole (or electron) scavengers is required to allow for the extraction of holes (or electrons) from the molecule complex^31^. Such phenomenon is also evidenced by the volume slices of calculated charge densities for the three models with COOH/HCOO adsorbate (Fig. 1g and Supplementary Fig. 5) and corresponding Bader charge analysis (Supplementary Table 3), which clearly depicted the weakest electronic density interaction of COOH with Sn–Bi interface model. Hence, bimetallic Sn–Bi interface suppresses the competing CO formation through weakening the COOH adsorption. Interestingly, the most active Sn–Bi interface site towards HCOOH formation delivers the moderate HCOO* adsorption, most likely arising from the absence of π backdonation between Sn–O interaction, which leads to positive dependence of *p*–*d* interaction on the electron density of the metal. Another possible explanation is that stronger adsorption of HCOO* intermediate of Sn–Bi alloy makes the breaking chemical bonds more difficult in the next elementary step, which follows the Brønsted–Evans–Polanyi (BEP) relations^16,37,38^.

In brief, the enhanced reactivity of Sn–Bi bimetallic interface can be ascribed to the following aspects as unveiled by the theoretical calculations: (i) Sn exhibits electron density depletion and owns the moderate valence electron bank by interacting Bi at the interface; (ii) the binding of COOH is weakened enabling suppressed CO formation; (iii) the affinity of HCOO is tuned by the optimum moderate adsorption, boosting HCOOH production.

### Synthesis and structural characterization

**Fig. 2:**
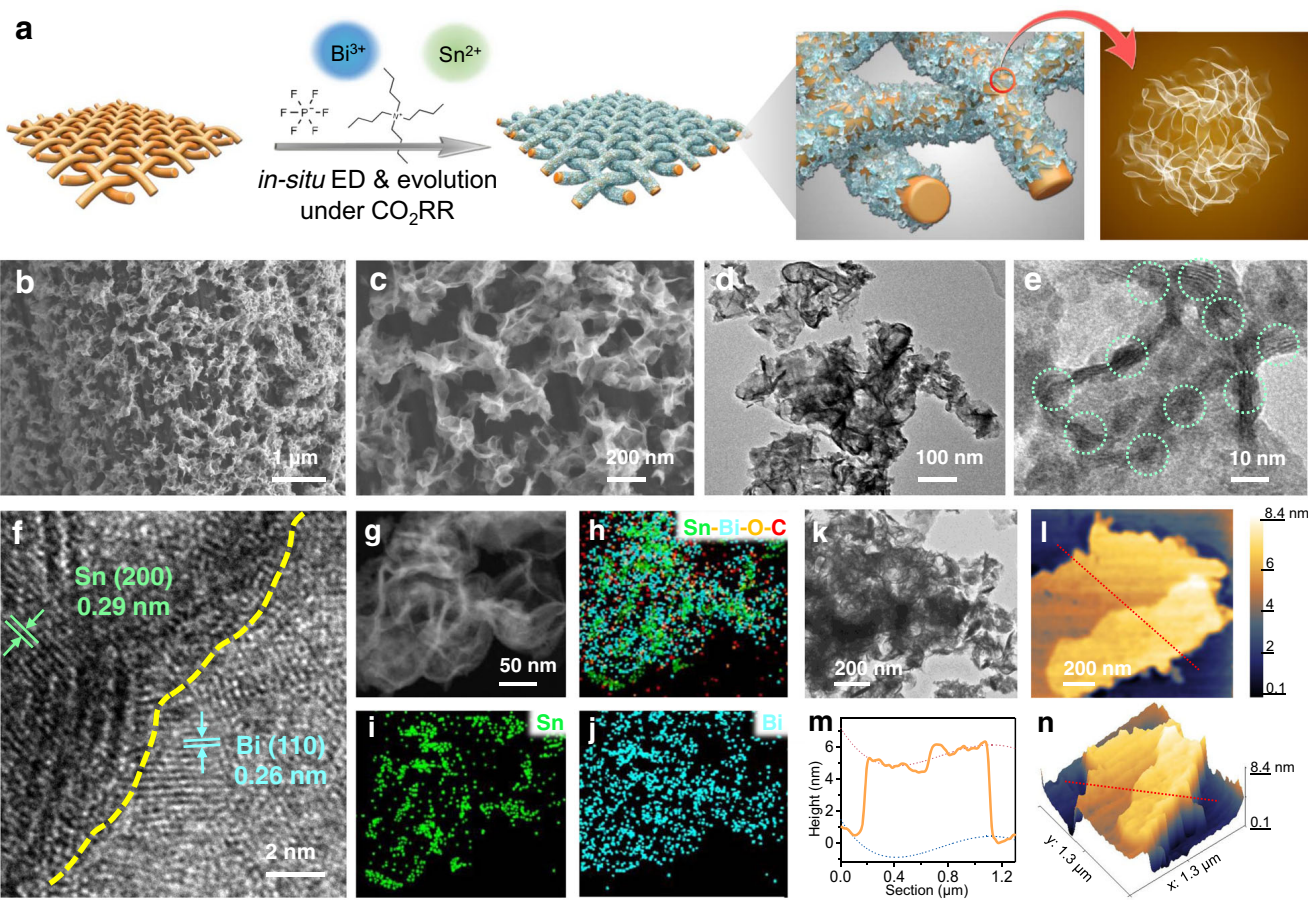
Fabrication and characterizations of Sn–Bi interface induced by crumples. **a** Scheme of the synthesis process through in situ electrodeposition and evolution under CO_2_RR conditions. **b**, **c** SEM images of microflower structures on CF. **d** TEM image of flower structures composed by nanosheets. **e**, **f** HRTEM images of nano-crumples on the flower petal. **g** HAADF-STEM image and (**h**–**j**) corresponding EDS element mapping. **k** TEM image of flower structures. **l** AFM image of the similar structure (**k**). **m**, **n** The corresponding height profile, and three-dimensional AFM image.

Inspired by these theoretical findings, we investigated a simplified method to expose abundant active Sn–Bi interface. As shown in Fig. 2a, in situ electrodeposition (ED) and evolution under CO_2_RR conditions were applied to purposefully expose Sn–Bi interfaces, which were induced by the steered crumple densities with precisely controlled ED parameters. The three-dimensional highly porous carbon fabrics (3D CF) were adopted as the substrates to disperse the catalytic sites in luxuriant and orthometric carbon fibers^39,40^. Such CF substrates were activated through C–O bonds by nitrate anions and immersed in the catalyst precursor (Sn^2+^ and Bi^3+^) for 2 days^41^, which were dispersed and stabilized by a complexing agent of tetrabutylammonium hexafluorophosphate^42^. Under cathodic potentials, Sn^2+^ and Bi^3+^ ions were reduced to metals in sequence on CF, accompanied by the current of CO_2_RR (Supplementary Fig. 6).

The dependency of the structures on constant current electrodeposition (CC-ED) and constant potential electrodeposition (CP-ED, Supplementary Fig. 7) was also investigated. The CC-ED can create uniform nanosheets with crumples, while CP-ED resulted in porous nanosheets or chunks on CF (Supplementary Fig. 8). The effects of CC-ED time and CO_2_ flow speed on crumples densities were further investigated in an attempt to gain insight into the growth of catalysts and find the best exposure of Sn–Bi interface during ED (Supplementary Fig. 9). It was found that the surface densities of crumples were higher with longer ED time and higher flow speed of CO_2_ from TEM images. However, large particles were also observed in SEM images, which made the synthesized materials not uniform. Additionally, there are no crumples with bubbling of Ar, indicating that the structural reconstruction induced by CO_2_RR remarkably affected the crumples formation and exposition of Sn–Bi interface.

Scanning electron microscopy (SEM) image of the as-prepared Sn–Bi bimetallic material showed that microflower morphology assembled from tortile nanosheets grow uniformly and oriented to different angles on the porous 3D CF substrate (Fig. 2b, c) with optimum ED parameters. To further investigate this microstructure, the high-resolution TEM (HRTEM) images were examined as revealed in Fig. 2d–f. The tortile bimetallic nanosheets were so thin that they were almost transparent to the electron beam (Fig. 2d). Also, the tortile nanosheets contain many local nano-wrinkle structures. As illustrated by the HRTEM and high-angle annular dark-field scanning TEM (HAADF-STEM) images (Fig. 2f, g), the nano-crumples structure exhibits a zone of bimetallic interfacial contact between Sn and Bi structures. The lattice fringes with a d-spacing of 0.29 nm and 0.26 nm correspond to the numerous step edges of Sn (200) plane and Bi (110) plane, respectively. Energy-dispersive X-ray spectroscopy (EDS) mapping (Fig. 2h–j) was conducted to confirm the Sn–Bi bimetallic interface structure.

Atomic force microscopy (AFM) was also performed to characterize the flower structures with crumples, and one TEM image with a similar structure is shown for comparison (Fig. 2k, l). The height profile and the 3D image revealed the crumple topology (Fig. 2m, n, Supplementary Fig. 10). Such images show clear crumpled roughness on the flower with a height of around 5 nm. The Sn–Bi interface is more active according to the DFT calculations, thus, the aforementioned Sn–Bi materials induced by crumples are expected to have higher activities towards CO_2_RR ascribed to the abundant active sites.

### Crystal structures and electronic structures analysis

**Fig. 3:**
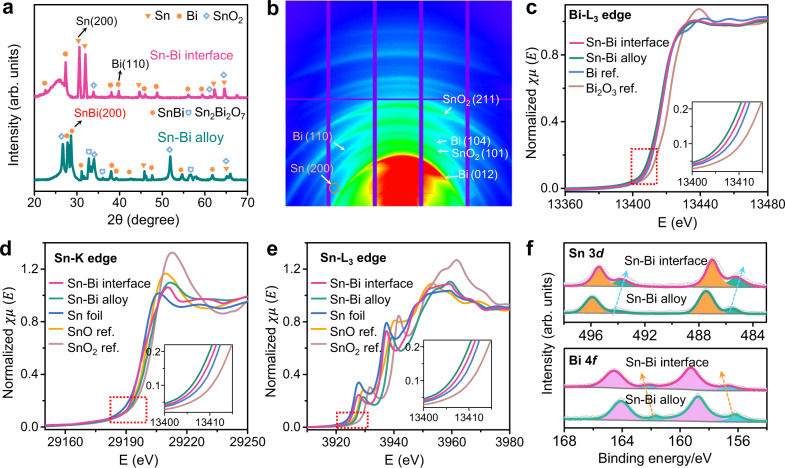
Crystal structures and electronic structures analysis. **a** XRD patterns. **b** Synchrotron 2D-XRD patterns of the Sn–Bi bimetallic interface. **c**–**e** XANES spectra of Bi L_3_-edge, Sn K-edge and Sn L_3_-edge. **f** XPS spectra of Sn 3d spectrum and Bi 4 f spectrum for Sn–Bi interface and alloy. Solid color lines represent the curve fittings.

The crystal structures and electronic structures analysis of the Sn–Bi bimetallic interface at the crumples and Sn–Bi alloy are shown in Fig. 3. A comparison of the X-ray diffraction (XRD) patterns of the Sn–Bi bimetallic interface and Sn–Bi alloy is displayed in Fig. 3a. The XRD pattern of the Sn–Bi bimetallic interface matches well with the peaks of Sn (tetragonal, JCPDS# 04-0673) and Bi (rhombohedral, JCPDS# 44-1246), which confirm the coexistence of Sn and Bi-metal phases with the predominant crystal planes of (200) and (110), respectively. The crystal planes distribution of aforementioned Sn–Bi bimetallic interface at the crumples was also identified by two-dimensional synchrotron X-ray diffraction (2D-XRD) with a beam size of ~3 × 6 μm, as shown in Fig. 3b. That result further confirmed that no bulk alloy peak was observed for the Sn–Bi bimetallic interface materials compared with the Sn–Bi alloy sample (Supplementary Fig. 11). These results reveal that negligible amounts of Bi atoms enter into the Sn lattice (and vice versa), thus almost no Sn–Bi bulk alloy was formed during the electrodeposition synthesis. Nevertheless, there is a distinct Sn–Bi (200) alloy peak located at 2θ of 29° for the Sn–Bi polyhedron materials fabricated by the hydrothermal method (tetragonal, JCPDS#27-0896, Supplementary Figs. 11–12), which was used as the reference of Sn–Bi bulk alloy in line with the DFT calculations. Because the Sn–Bi binary material by in situ ED under CO_2_RR method preferably forms the Sn–Bi interface or surface alloy structure, the hydrothermal method was adopted to fabricate the Sn–Bi bulk alloy structure to compare the aforementioned Sn–Bi bimetallic interface material. Synchrotron 2D-XRD pattern (Supplementary Fig. 11c) further confirmed the existence of Sn–Bi (200) crystal planes together with crystals of SnO_2_ and Bi_2_O_3_, which were more likely due to the oxidation process in hydrothermal procedure^14^. It was also observed that the Bragg reflections of Sn–Bi (200) alloy peak shift to lower 2θ angles, indicating the linear expansion of the tetrahedral Sn lattice due to the addition of larger Bi atoms into Sn (lattice mismatch >22%)^23,43^.

X-ray absorption spectroscopy (XAS) was conducted to investigate the electronic structures of Sn–Bi materials. Figure 3c–e depicted the X-ray Absorption Near-edge Fine Structure (XANES) spectra of Bi L_3_-edge, Sn K-edge, and Sn L_3_-edge respectively, which reveal the pre-edge features based on the absorption edge or the location of white lines. In general, the shapes of the Bi L_3_-edge for the Sn–Bi bimetallic interface and alloy are close to that of the Bi foil (Fig. 3c) whereas the white line intensity at ~13,440 eV is much weaker than the reported Bi_2_O_3_^44^. The latter implies that the Bi in the materials is mainly in the metallic state. A negative shift of the absorption edge position was found for the Bi L_3_-edge after interacting Sn elements compared with that of Bi foil (Fig. 3c), illustrating the electron density transfer from Sn to Bi. This is also confirmed by the opposite shift of Sn K-edge with Bi elements introduced (Fig. 3d). The intensity of the white line (2*p* → 5*d* orbital transition) in the XANES at Bi L-edge of Sn–Bi alloy is slightly lower than that of the Sn–Bi bimetallic interface, which can be attributed to more filling of the Bi 5*d* bands due to alloying with Sn. This implies the moderate electron transfer in the Sn–Bi interface compared with pure Sn and Sn–Bi alloy, which is also consistent with previous DFT results. In addition, these findings were also observed in Fig. 3d, e with the evidence of a more positive shift and higher intensity of white line of Sn K-edge and L_3_-edge in Sn–Bi alloy. A slight lowering of the white line peak intensity of Sn K-edge for the Sn–Bi bimetallic interface compared to the Sn–Bi alloy (Fig. 3d), also indicates a less oxidized state of Sn in the bimetallic interface structure.

X-ray photoelectron spectroscopy (XPS) was conducted to study the surface oxidation states of Sn and Bi elements in Sn–Bi materials (Fig. 3f and Supplementary Fig. 13). The peaks located at 493.6 eV and 485.2 eV corresponds to metallic Sn 3*d*_3/2_ and 3*d*_5/2_ for Sn–Bi interface material, while the appearances of peaks at 495.4 eV and 487.0 eV are mainly fitted to Sn^2+/4+ ^^8,14^. This indicates that the surface Sn was partially oxidized in a short time when exposed to the air. The XPS spectra of Sn in the Sn–Bi interface shift to the lower energy compared to that of Sn–Bi alloy, implying the lower average oxidation state of Sn atoms, which is attributed to the less electron transfer from Sn to Bi atoms in the Sn–Bi interface. The peaks at 156.4 eV and 161.9 eV of Sn–Bi interface material are assigned to metallic Bi 4*f*_7/2_ and Bi 4*f*_5/2_, respectively, whereas the Bi 4*f*_7/2_ and Bi 4f_5/2_ peaks at 157.7 and 163.2 eV, respectively. These are also ascribed to the surface oxidation of Bi in air^45^. In comparison with Sn–Bi alloy, the XPS spectra shift to the higher energy for the Sn–Bi interface material, indicating the slightly higher oxidation state of Bi atoms. These results observed in XPS illustrate the electron density depletion of Sn and accumulation of Bi in Sn–Bi materials, and further elucidate the moderate electron transfer within Sn–Bi interface material compared with those of their counterpart pure metal and alloy structure. This observation is also consistent with DFT simulations and XAS analysis.

### In situ extended X-ray absorption fine structure and Raman analysis

**Fig. 4:**
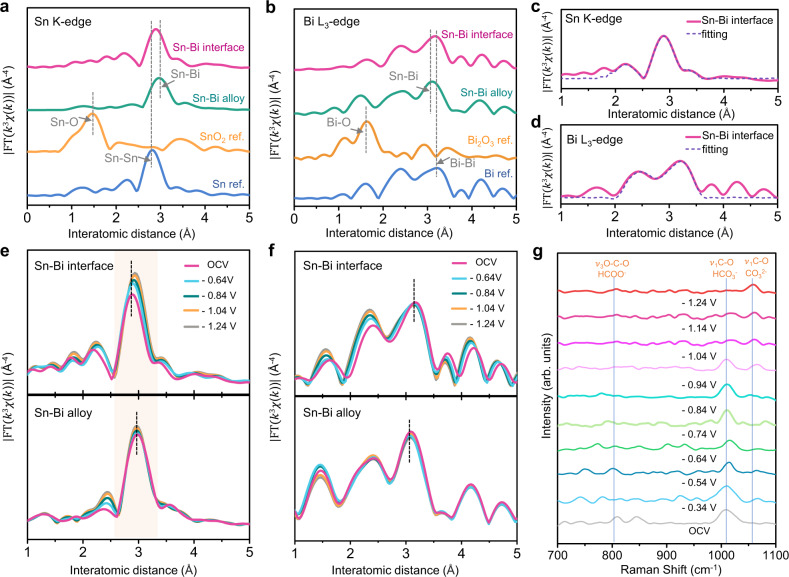
Characterizations of local atomic structures and the chemical environment by EXAFS. **a**, **b** Fourier-transform k^3^-weighted EXAFS spectra of Sn K-edge and Bi L_3_-edge of Sn–Bi alloy, bimetallic interface materials and reference samples. **c, d** Representative fitting of the EXAFS spectra to the R space of Sn K-edge and Bi L_3_-edge of Sn–Bi bimetallic interface materials. **e, f** In situ Fourier-transform k^3^-weighted EXAFS spectra for Sn K-edge and Bi L_3_-edge of Sn–Bi interface and alloy samples, respectively, at different applied potentials during CO_2_RR. **g** In situ Raman spectroscopic study on the intermediate adsorption for Sn–Bi interface sample at various potentials vs. RHE.

In situ extended X-ray absorption fine structure (EXAFS) was performed under operating conditions at various applied voltages to investigate the local atomic structures and chemical environment (Fig. 4). In the Sn K-edge EXAFS spectra (Fig. 4a), metallic Sn^0^ and Sn^2+/4+^ were characterized by employing Sn and SnO_2_ as references respectively, where the peaks located at 1.42 Å and 2.80 Å correspond to Sn–O and Sn–Sn bonds^46^. The one prominent peak observed at around 2.87 Å can be attributed to the combined scattering path of Sn–Sn and Sn–Bi bonds^45,47^. Likewise, a dominant peak appearing at a similar position with the Bi–Bi bond of Bi reference was detected and identified as the combined scattering path of Bi–Bi and Sn–Bi bonds (Fig. 4b). This is owing to that the existence of Bi triggers the electron depletion of surface Sn atoms, potentially building the interaction between the electron density of these two elements, which leads to a trace amount of Sn–Bi bonds. Moreover, the Sn–Bi bond length lies between the Sn–Sn and Bi–Bi bonds. Representative fitting of the EXAFS spectra to the R space of Sn–Bi bimetallic interface and alloy materials are shown in Fig. 4c, d and Supplementary Fig. 14. Sn K-edge and Bi L_3_-edge EXAFS spectra fit simultaneously with constraints employed to ensure that the bond lengths and disorder factors for Sn–Bi and Bi–Sn bonds are the same. The corresponding fittings to the k space are depicted in Supplementary Fig. 15. The fitting results of in situ FT-EXAFS for Sn–Bi interface and alloy are summarized in Supplementary Table 4 and Table 5. Compared with Sn–Bi alloy, Sn–Bi bimetallic interface has higher coordination numbers (CN) of Sn–Sn and Bi–Bi but lower CN of Sn–Bi. This confirms the segregation nature of Sn–Bi bimetallic interface structure compared to Sn–Bi bulk alloy structure, consistent with the previous characterization. Larger Bi atoms result in lower CN with Sn (e.g., total CN of 3.4 for Sn in Sn–Bi alloy) compared to that of Sn–Sn (CN = 4) in Sn foil reference^23,48^. Wavelet transform EXAFS was also performed with high resolution in both k and R space (Supplementary Fig. 16). The strong WT signal of Sn–Bi and Sn–Sn bonds were visible, compared to the SnO_2_ reference counterpart.

Upon application of potential from an open circuit potential (OCV) to −1.24 V (vs. RHE) during CO_2_RR, the Sn–Bi bimetallic interface/alloy structure can be well maintained, indicated by the little difference of in situ Fourier-transform EXAFS spectra for Sn K-edge (Fig. 4e) and Bi L_3_-edge (Fig. 4f). The fitting results of Sn K-edge show that the main peaks tend to slightly shift to a longer bond length when decreasing the potentials to −1.24 V (Supplementary Table 4). Considering that an increase in Sn–Sn coordination numbers is paralleled by a decrease in Sn–Bi coordination numbers, it is likely that further dealloying of Sn–Bi phase takes place (i.e., further segregation of Sn and Bi). The fitting of in situ EXAFS of Bi L_3_-edge also shows the increased distortion of Bi–Sn and Bi–Bi bonds with the cathodic voltages decreasing. In situ XANES spectra of Sn K-edge and Bi L_3_-edge of Sn–Bi interface and alloy samples at different applied potentials are presented in Supplementary Figs. 17–18. The slight lowering of the white line peak intensity and lower-energy shift of absorption edge when decreasing the potentials indicate the electron density accumulation resulted from the slight reduction of Sn and Bi under operating conditions^14,49^. The XAS data acquisition details and in situ liquid cell used for measurements (Supplementary Fig. 19) are described in the Supplementary Information.

In situ Raman spectroscopy studies on the intermediate adsorption for Sn–Bi interface samples at various potentials were carried out (Fig. 4g) to show the mechanism of surface chemical environments under different potentials. The characteristic Raman bands can be assigned to the corresponding adsorbed surface intermediates^50–52^: (1) symmetric C–O stretching vibration of HCO_3_‾ (*ν*_1_, 1010–1020 cm^−1^), (2) symmetric C–O stretching vibration of CO_3_^2^‾ (*ν*_1_, 1060–1070 cm^−1^), (3) *ν*_3_ mode of HCOO^−^, i.e., symmetric O–C–O bending (scissor) mode (796–802 cm^−1^). Obviously, with different applied potentials, the distributions of adsorbed intermediate species are different. Moreover, with the increase of potential, the prominent HCO_3_‾ peak gradually disappeared while the CO_3_^2−^ peak became dominant starting from −0.84 V vs. RHE. This observation might be caused by: (i) a rise in local pH values^29^; (ii) the equilibrium exchange between bicarbonate (CO_3_H*) with dissolved CO_2_, which affects the transport of CO_2_ across the double layer^53^.

### Electrochemical CO_2_ reduction performance

**Fig. 5:**
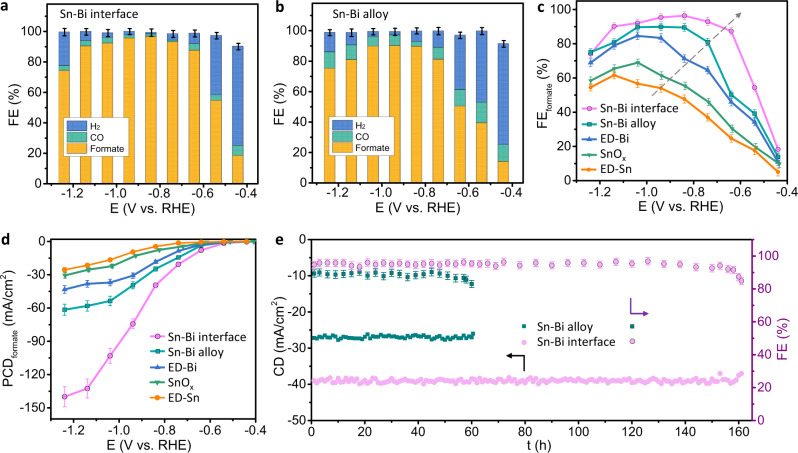
CO_2_RR performance. **a**, **b** FE toward CO, formate and H_2_ for Sn–Bi interface and Sn–Bi alloy. **c** FE of formate, **d** Partial current density toward formate for various samples: Sn–Bi interface, Sn–Bi alloy, ED-Bi from electrodeposition, SnO_x_ from hydrothermal method, and ED-Sn from electrodeposition. **e** The stability testing of Sn–Bi interface and alloy at −0.84 V vs. RHE.

The catalytic performance of Sn–Bi bimetallic interface and Sn–Bi alloy for CO_2_RR was evaluated in an H-cell with CO_2_-purged 0.5 M KHCO_3_ as the electrolyte (Fig. 5). Linear sweep voltammetry (LSV) was conducted in the CO_2_- and Ar-purged catholyte to initially evaluate the CO_2_RR performance (Supplementary Fig. 20). Accordingly, the gaseous and liquid products were quantitatively analyzed via online gas chromatograph (GC) and ^1^H nuclear magnetic resonance (^1^H NMR) spectroscopy, respectively. Sn–Bi interface material demonstrates a remarkable maximum formate Faradaic efficiency (FE) of 96.4 ± 2.5% at a low potential of −0.84 V vs. RHE (Fig. 5a), while Sn–Bi alloy shows a lower maximum FE_formate_ of 89.9 ± 2.5% at a higher potential of −0.94 V vs. RHE (Fig. 5b). The FE_formate_ is maintained over 90% at a wide potential window (−0.74 to −1.14 V vs. RHE), which might be attributed to the efficient mass transport of porous microflower structures, ensuring the desired local environment near the active sites^28,54^. This reveals that the crumples induced Sn–Bi interface material achieves a higher selectivity of formate at a lower overpotential. For comparison, SnO_x_, electrodeposited Sn and Bi were also synthesized and evaluated to further manifest the synergistic effects of Sn and Bi at the electrochemical interfaces.

Comparing various catalysts toward formate formation (Fig. 5c, Supplementary Fig. 21), a notable improvement of FE_formate_ is obtained after involving Bi at the interface of pure Sn. In addition, the overpotential of achieving the maximum FE_formate_ decreases. It is attributed that Sn tends to transfer electrons from the *p* orbital to the interface Bi atoms and owns the moderate valence electron bank compared to pure Sn and Sn–Bi alloy, which best balances the adsorption of two competing HCOO and COOH intermediates (Supplementary Table 2) and delivers the best selectivity of formate. As exhibited in Fig. 5d, the highest HCOOH partial current density (PCD) is reached by the Sn–Bi interface material compared to those of pure Sn, Bi, SnO_x_, and Sn–Bi alloy. More importantly, to the best of our knowledge, the PCD_formate_ outperforms most of the reported Sn-based and Bi-based catalysts at low potentials (−0.74 to −1.14 V vs. RHE) with high FE_formate_ (>90%) (Supplementary Table 6). The PCD_formate_ of this work at −0.94 V is also comparable with one of the highest reported performances using three-dimensional bismuthene catalyst^55^. A superior PCD_formate_ of 140 mA/cm^2^ is achieved at −1.24 V vs. RHE. This is attributed to the enriched active Sn–Bi interface sites created by the abundant crumples.

Long-term performance is another feature introduced by catalysts developed in this work. The stability testing was evaluated at a constant potential of −0.84 V and FE_formate_ at around 90% (Fig. 5e), Sn–Bi interface material presents a stable current density of around 40 mA/cm^2^ and FE_formate_ of over 90% for around 160 h with negligible degradation (Supplementary Figs. 22–23), which far exceeds other recently reported data of Sn-based or Bi-based materials^23,26,56^. The HAADF-STEM and EDS mapping also depicted the stable existence of Sn–Bi interface after the long-term stability test (Supplementary Fig. 24). This demonstrates that this material by in situ electrodeposition under CO_2_RR and ligand modification is highly stable during CO_2_RR. This is benefited from well-defined surface structures at steady crumples fabricated by the controlled ED method. On the other hand, Sn–Bi alloy fabricated by the hydrothermal method holds lower stability (around 60 h) with a constant current density of 28 mA/cm^2^ compared with the electrodeposited Sn–Bi interface, revealing the simultaneous improvements in terms of activity and stability. After the stability test, the Sn–Bi alloy maintains the phases as confirmed by SEM images, TEM images, and EDS element mapping (Supplementary Figs. 23, 25).

**Fig. 6:**
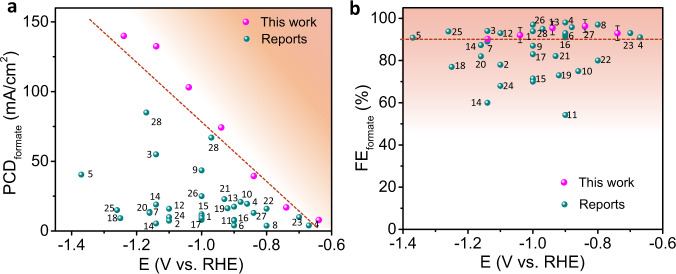
CO_2_RR performance comparison with the reported data in the literature. **a** PCD toward formate and **b** FE for formate. (The inserted reference numbers represent the references in Supplementary Table 6 in the Supplementary Information).

We report a method of in situ electrodeposition to expose the active Sn–Bi interface and reveal the correlation between the catalytic activities of Sn–Bi system with their bimetallic patterns, as well as the evolutions of their local atomic structures and chemical environment during CO_2_RR, which have not been discussed in the other reported Sn–Bi alloy or oxides system^14,23,26,57,58^ to the best of our knowledge. A distinct comparison of our work with those in the literature is also illustrated in Fig. 6. The reported best FE_formate_ during CO_2_RR and corresponding PCD_formate_ are summarized for the present Sn-based and Bi-based catalysts. It is shown that the PCD_formate_ of the synthesized materials surpasses almost all the currently reported data in the literature at all operating voltages (Fig. 6a). Figure 6b displays the comparison of FE_formate_. As shown in this figure, the corresponding FE maintains over 90% at a wide potential window (−0.74 to −1.14 V vs. RHE) and is at the top array among the published data.

Given the performance advance of this study compared with the literature, we attribute the >100 mA/cm^2^ CO_2_RR current density to the intrinsic activity improvements of abundant crumple-induced Sn–Bi interface active sites by in situ electrodeposition under CO_2_RR conditions. We prove experimentally and theoretically that the fine-tuned exposing Sn–Bi interface is the most favorable structure for HCOOH formation compared to pure Sn and Sn–Bi bulk alloy. In addition, the nano-crumple-induced Sn–Bi interface material on carbon fabric substrate ensures rapid mass/charge transport hierarchically. Firstly, it has microflower morphology assembled from tortile nanosheets, growing tridimensionally and orienting to different angles, which provides multiple interconnected channels^55^. Secondly, the porous three-dimensional carbon fabric employed in this work not only ensures a uniform distribution of active Sn–Bi interface material but also endows with interlaced channels for mass and charge transfer on the microscale.

The CO_2_RR was also tested in a homemade GDE flow cell (Supplementary Fig. 26), which shows the performance of ~320 mA/cm^2^ at 1.20 V vs. RHE using neutral electrolyte (0.5 M KHCO_3_), resulting from better mass transportations. The performance comparisons of flow cells in this study with those in the literature are listed in Supplementary Table 7. To reflect the intrinsic activity of a catalyst, we evaluated the electrochemically active surface area (ECSA) measurements and normalized current density plots as well (Supplementary Figs. 27–29). The double-layer capacitance and the corresponding ECSA are summarized in Supplementary Table 8.

## Discussion

In this study, we report the fine control of overpotential electrodeposition under in situ CO_2_RR can be used to achieve an abundantly exposed active Sn–Bi interface pattern induced by nano-crumples morphology, which offers an attractive route to improve current densities at low potentials for formate production. As revealed by DFT calculations, XPS and in situ XAS characterizations, Sn–Bi bimetallic interface delivers the optimum upshift of the p-band center of Sn, and accordingly the moderate valence electron depletion, which leads to fragile adsorption of COOH and moderate adsorption of HCOO. Thus, with a lower thermodynamic barrier of HCOOH formation, a superior partial current density for formate is obtained (up to 140 mA/cm^2^). High FE_formate_ (>90%) is maintained at a wide potential window (−0.74 to −1.14 V vs. RHE) with longer durability (160 h) than those reported in previous studies. This design concept can also be extended to the highly active and stable interface design of other bimetallic catalytic systems.

## Methods

### Computational details

Density functional theory (DFT) calculations were realized via Vienna Ab Initio Simulation Package (VASP)^59^, version 5.4.4. The projector-augmented wave (PAW) framework was employed to calculate the interactions between valence electrons and ion cores^60^. The effect of electron exchange-correlation was estimated through Perdew-Burke-Ernzerhof functional based on generalized gradient approximation, known as PBE-GGA^61^. For all the structural relaxations, the convergence criteria include 1 × 10^−4^ eV for self-consistency loop of electronic structure, 400 eV energy cutoff for the plane-waves, and 0.01 eV/Å for Hellmann-Feynman^62^ force. We also adopted spin-polarization calculations, 2 × 2 × 1 Monkhorst-Pack k-points, and Gaussian smearing method (σ = 0.1 eV). The change in adsorption energy (<0.01 eV) was negligible when improving the calculation accuracy, i.e., an energy cutoff of 450 eV or k-points of 4 × 4 × 1. In this work, van der Waals interaction was considered to incorporate the long-range dispersion effects by implementing DFT-D2 method proposed by Grimme et al.^63^. A vacuum layer of 15 Å was implemented to avoid periodic interactions of neighboring slabs. Three surface models were built for DFT calculations (Supplementary Fig. 1)^14,16,23–26^. The detailed definitions of adsorption energy (*E*_*ads*_) are shown elsewhere^24,64,65^. The energetic data was corrected by zero-point energy (ZPE).

Gibbs free energies (G) were calculated as:

1 $${{{{{\rm{G}}}}}}={E}_{{{{{{\rm{DFT}}}}}}}+{E}_{{{{{{\rm{ZPE}}}}}}}-{{{{{\rm{TS}}}}}}$$

where *E*_DFT_ represents the total energy calculated from DFT, *E*_ZPE_ is the vibrational ZPE. T (system temperature) is 298.15 K. *E*_ZPE_ and entropy (S) of adsorbates were calculated based on vibrational frequencies calculations, while NIST database^66^ was used for those of molecules. We considered the dipole and solvent corrections with the same settings in the references^56,64^.

We calculate the density of states (DOS) calculations by utilizing a more accurate k-points mesh of 8 × 8 × 1. The volume slice of charge density analysis was visualized by VESTA (https://jp-minerals.org/vesta/en/) through electron density calculation from DFT. The miller indices of the cutoff two-dimensional visualization surface are 100, which exposes the side view of the adsorbates and surface models. Bader charges of all atoms were studied through Bader Charge Analysis code from Henkelman’s group^67^. The formal charge is defined as atomic Bader partial atomic charges subtracted from the corresponding valence electrons of the elements.

### Crumpled Sn–Bi interface preparation

3D porous conductive carbon fabric (Fuel Cell Earth) was employed as the substrate to grow Sn–Bi interface structures, which was pretreated by consecutive ultrasonication in distilled deionized (DDI) water, acetone, and ethanol. Then it was activated by immersing into HNO_3_ solution (65 wt%) overnight^68^. The catalyst precursor was composed of 2 mL of 0.1 M Tin(II) acetate (Alfa) solution, 2 mL of 0.1 M Bismuth(III) chloride (Sigma–Aldrich) solution, and 6 mL of 0.02 M Tetrabutylammonium hexafluorophosphate (Sigma–Aldrich) solution, then pH value was adjusted by adding hydrochloric acid (HCl, 37%, Sigma–Aldrich) until the solution was transparent. Several pieces of pretreated CF were firstly immersed in this precursor for 48 h to be fully wetted. Such CF was then employed as the working electrode, whereas a saturated calomel electrode (SCE) as the reference electrode and platinum foil as the counter electrode. The precursor was added into 40 mL CO_2_ saturated 0.1 M KHCO_3_ (Sigma–Aldrich) solution as the electrolyte for the electrodeposition. The electrodeposition was optimized to constant current electrodeposition at 15 mA/cm^2^ for 30 min through a BioLogic VSP300 potentiostat with continuous CO_2_ bubbling at a speed of 20 sccm. After the electrodeposition process, the obtained crumpled Sn–Bi interface on CF was rinsed repeatedly with DDI water and ethanol, and then dried at 80 °C in a vacuum oven overnight. The loading of catalysts on CF was about 1.0 mg/cm^2^ by weighing the CF before and after the deposition step. The ED-Bi and ED-Sn samples were synthesized by CC-ED with the metal precursor composed of Tin(II) acetate solution and Bismuth(III) chloride solution, respectively. The densities of crumples were controlled by different times of CC-ED and flow speed of CO_2_ during in situ ED and evolution.

### Sn–Bi alloy preparation

Sn–Bi alloy structure was directly grown on the CF substrate by the hydrothermal method^14,68^. Specifically, 0.143 g of Tin (II) acetate (Alfa), and 0.190 g of Bismuth (III) chloride (Sigma–Aldrich), 0.073 g of urea (Sigma–Aldrich), and 0.112 g of ammonium fluoride (Sigma–Aldrich) were transferred in 60 mL DDI water by vigorous stirring. The pH values were tuned by hydrochloric acid until the solution turned transparent. Such precursor solution along with one piece of pretreated CF (2.5 × 5.0 cm^2^) was transferred into a 100 mL Teflon-lined autoclave and heated to 180 °C for 10 h. Sn–Bi alloy on CF obtained from the hydrothermal process was rinsed repeatedly with DDI water and ethanol, then dried overnight at 80 °C under vacuum. The loading of Sn–Bi alloy on CF was also around 1.0 mg/cm^2^ by weighing the CF before and after the hydrothermal procedure. SnO_x_ sample was also obtained by this hydrothermal method with a metal precursor composed of only Tin (II) acetate.

### Characterization

The as-prepared materials were characterized by XRD (Rigaku Miniflex 600) to disclose crystal structures. XPS (Thermo Scientific K-Alpha XPS spectrometer) was employed to reveal elemental compositions and oxidation states. SEM (LEO FESEM 1530 and Oxford Ultim Extreme) and TEM (JEM 2100 F) were employed to observe the morphologies and microstructures. High-resolution TEM and scanning TEM were conducted using a high-brightness electron source and an aberration-correcting transmission electron microscope, the FEI Titan 80–300 (FEI Company, The Netherlands) equipped with a Gatan Quantum energy filter (Gatan Inc., USA). An annular dark-field detector was used for image acquisition.AFM was performed by Dimension ICON with Nano Scope V controller (Bruker) in Scan Asyst and approaching mode.

The synchrotron 2D-XRD images were performed on VESPERS beamline at the Canadian Light Sources. In situ and ex situ XANES and EXAFS of Sn K-edge and Bi L_3_-edge were acquired on HXMA beamline at Canadian Light Source Inc, Canada, and BL11B beamline at Shanghai Synchrotron Radiation Facility, China. Ex situ XANES of Sn L_3_-edge was acquired on SXRMB beamline at Canadian Light Source Inc, Canada. The in situ CO_2_RR experiments were performed immediately after the electrodeposition step to avoid the oxidation of electrodes in the air. The XANES and EXAFS results were analyzed via Athena and Artemis software packages (http://bruceravel.github.io/demeter).

In situ Raman Spectroscopy was conducted by an in situ Raman custom-made flow cell with a working electrode of 0.5 cm^2^ and a CO_2_-purged 0.5 M KHCO_3_ electrolyte. A laser confocal microspectrometer (Renishaw inVia) with a 532 nm wavelength laser was utilized to collect Raman spectrum. To obtain a clear analysis, the background of raw data within 700–1100 cm^−1^ was processed via automatic intelligent fitting-mode baseline subtraction by WiRE software.

### Electrochemical measurement

According to our previous work^14^, CO_2_RR experiments were performed in an H-cell and both catholyte and anolyte were 0.5 M KHCO_3_ solution (30 ml). The as-prepared materials were used as the working electrode, while the reference electrode was SCE, and the counter electrode was platinum foil. The catholyte was continuously bubbled with CO_2_ for 30 min before the measurement to make it CO_2_-saturated. CO_2_RR tests were carried out through the chronoamperometry technique at constant potentials ranging from −0.44 to −1.24 V (vs. RHE) for 1 h at each potential using a BioLogic VSP300 potentiostat. Throughout this study, current densities were normalized to the geometric surface area. The results presented are the averaged values with error bars from three independent measurements. All the experiments were performed under ambient pressure and at room temperature (25 °C). The Chronoamperometry technique was also employed in the stability measurement, and the potential was set to −0.84 V vs. RHE. The electrolyte samples (800 μL each) were collected every 3 h in the first three days and sealed for further NMR analyses. Gas products (CO and H_2_) were analyzed by the online GC. Then the products were analyzed every 6 h until FE_formate_ dropped under 90%. To eliminate the effect of produced formate accumulation in the electrolyte, the electrolyte was renewed every 24 h^55^. The stability tests were repeated three times.

### Products analysis

The analyses of products were the same as our lab reported^28^. The concentration of liquid products, especially formate in this work, in the catholyte was quantified by a 500 MHz ^1^H liquid NMR spectrometer (Bruker Advance) with the water suppression method^14^. Gas products, such as CO and H_2_, were quantified using online gas chromatography (GC, SRI 8610 C) with a packed Molecular Sieve column, Helium ionization detector (HID), and the carrier gas of Helium (Praxair Gas, 99.999%).

### Faradaic efficiency and partial current density

The Faradaic efficiency (FE) can be calculated as the following equation:

2 $${{{\mathrm{FE}}}}=\frac{{znF}}{Q}$$

where, $z$ is the number of electrons exchanged in the reaction (two-electron transfer for formate and CO production), $n$ is the number of moles of product formed, $F$ is the Faraday constant, and $Q$ is the total amount of charges passed through the CO_2_RR process. The partial current density was then obtained by multiplying the Faradaic efficiency with the average current density.

## Supplementary information

Supplementary Information

## Data Availability

All data needed to evaluate the conclusions in the paper are present in the paper and/or the Supplementary Materials.

## Code availability

VESTA (https://jp-minerals.org/vesta/en), Bader Charge Analysis code (https://theory.cm.utexas.edu/henkelman/research/bader), and Athena software (http://bruceravel.github.io/demeter) are all open-source packages.

## References

[1] Lee JH (2019). Tuning the activity and selectivity of electroreduction of CO_2_ to synthesis gas using bimetallic catalysts. Nat. Commun..

[2] Wang S, Guan BY, Lou XW (2018). Rationally designed hierarchical N-doped carbon@NiCo_2_O_4_ double-shelled nanoboxes for enhanced visible light CO_2_ reduction. Energy Environ. Sci..

[3] Shi R (2020). Efficient wettability-controlled electroreduction of CO_2_ to CO at Au/C interfaces. Nat. Commun..

[4] Zhong M (2020). Accelerated discovery of CO_2_ electrocatalysts using active machine learning. Nature.

[5] Cano ZP (2018). Batteries and fuel cells for emerging electric vehicle markets. Nat. Energy.

[6] Garcia de Arquer FP (2020). CO_2_ electrolysis to multicarbon products at activities greater than 1 A cm^-2^. Science.

[7] Xia C (2019). Continuous production of pure liquid fuel solutions via electrocatalytic CO_2_ reduction using solid-electrolyte devices. Nat. Energy.

[8] Luc W (2017). Ag-Sn bimetallic catalyst with a core-shell structure for CO_2_ reduction. J. Am. Chem. Soc..

[9] Zhang W (2018). Electrochemical reduction of carbon dioxide to methanol on hierarchical Pd/SnO_2_ nanosheets with abundant Pd–O–Sn interfaces. Angew. Chem. Int. Ed..

[10] Lu Z (2019). An isolated zinc-cobalt atomic pair for highly active and durable oxygen reduction. Angew. Chem. Int. Ed..

[11] Wang Y (2019). Ensemble effect in bimetallic electrocatalysts for CO_2_ reduction. J. Am. Chem. Soc..

[12] Bai X (2017). Exclusive formation of formic acid from CO_2_ electroreduction by a tunable Pd-Sn alloy. Angew. Chem. Int. Ed..

[13] Kortlever R, Peters I, Koper S, Koper MTM (2015). Electrochemical CO_2_ reduction to formic acid at low overpotential and with high Faradaic efficiency on carbon-supported bimetallic Pd–Pt nanoparticles. ACS Catal..

[14] Wen G (2018). Orbital interactions in Bi-Sn bimetallic electrocatalysts for highly selective electrochemical CO_2_ reduction toward formate production. Adv. Energy Mater..

[15] Giannakakis G, Flytzani-Stephanopoulos M, Sykes ECH (2019). Single-atom alloys as a reductionist approach to the rational design of heterogeneous catalysts. Acc. Chem. Res..

[16] Ren W (2021). Isolated copper-tin atomic interfaces tuning electrocatalytic CO_2_ conversion. Nat. Commun..

[17] Morales-Guio CG (2018). Improved CO_2_ reduction activity towards C_2+_ alcohols on a tandem gold on copper electrocatalyst. Nat. Catal..

[18] Ross MB (2017). Tunable Cu enrichment enables designer syngas electrosynthesis from CO_2_. J. Am. Chem. Soc..

[19] Back S, Kim J-H, Kim Y-T, Jung Y (2016). Bifunctional interface of Au and Cu for improved CO_2_ electroreduction. ACS Appl. Mater. Interfaces.

[20] Zheng J (2021). Regulating electrodeposition morphology in high-capacity aluminium and zinc battery anodes using interfacial metal–substrate bonding. Nat. Energy.

[21] Zhao Y (2020). Surface reconstruction of ultrathin palladium nanosheets during electrocatalytic CO_2_ reduction. Angew. Chem..

[22] Wang Y (2020). Catalyst synthesis under CO_2_ electroreduction favours faceting and promotes renewable fuels electrosynthesis. Nat. Catal..

[23] Yang Q (2020). Novel Bi-doped amorphous SnO_x_ nanoshells for efficient electrochemical CO_2_ reduction into formate at low overpotentials. Adv. Mater..

[24] Ren B, Dong X, Yu Y, Wen G, Zhang M (2017). A density functional theory study on the carbon chain growth of ethanol formation on Cu-Co (111) and (211) surfaces. Appl. Surf. Sci..

[25] Woodruff, D. *Surface Alloys and Alloy Surfaces*. (Elsevier, 2002).

[26] Ma T (2021). Engineering Bi-Sn interface in bimetallic aerogel with 3D porous structure for highly selective electrocatalytic CO_2_ reduction to HCOOH. Angew. Chem. Int. Ed..

[27] Zhang Z (2021). “Two Ships in a Bottle” design for Zn–Ag–O catalyst enabling selective and long-lasting CO_2_ electroreduction. J. Am. Chem. Soc..

[28] Wen G (2020). Ternary Sn-Ti-O electrocatalyst boosts the stability and energy efficiency of CO_2_ reduction. Angew. Chem. Int. Ed..

[29] Birdja YY (2019). Advances and challenges in understanding the electrocatalytic conversion of carbon dioxide to fuels. Nat. Energy.

[30] Jiao Y, Zheng Y, Davey K, Qiao S-Z (2016). Activity origin and catalyst design principles for electrocatalytic hydrogen evolution on heteroatom-doped graphene. Nat. Energy.

[31] Rao VG, Aslam U, Linic S (2018). Chemical requirement for extracting energetic charge carriers from plasmonic metal nanoparticles to perform electron-transfer reactions. J. Am. Chem. Soc..

[32] Hassan FM (2015). Evidence of covalent synergy in silicon–sulfur–graphene yielding highly efficient and long-life lithium-ion batteries. Nat. Commun..

[33] Hammer B, Morikawa Y, Nørskov JK (1996). CO chemisorption at metal surfaces and overlayers. Phys. Rev. Lett..

[34] Sullivan, B. P., Krist, K. & Guard, H. *Electrochemical and Electrocatalytic Reactions of Carbon Dioxide*. (Elsevier, 2012).

[35] Shi R (2021). Room-temperature electrochemical acetylene reduction to ethylene with high conversion and selectivity. Nat. Catal.

[36] Cable JW, Sheline RK (1956). Bond hybridization and structure in the metal carbonyls. Chem. Rev..

[37] Zhao Z-J (2019). Theory-guided design of catalytic materials using scaling relationships and reactivity descriptors. Nat. Rev. Mater..

[38] Ferrin P (2009). Modeling ethanol decomposition on transition metals: a combined application of scaling and Brønsted− Evans−Polanyi relations. J. Am. Chem. Soc..

[39] Xing Z, Hu L, Ripatti DS, Hu X, Feng X (2021). Enhancing carbon dioxide gas-diffusion electrolysis by creating a hydrophobic catalyst microenvironment. Nat. Commun..

[40] Yang H (2020). Carbon dioxide electroreduction on single-atom nickel decorated carbon membranes with industry compatible current densities. Nat. Commun..

[41] Zheng J (2021). Regulating electrodeposition morphology in high-capacity aluminium and zinc battery anodes using interfacial metal–substrate bonding. Nat. Energy.

[42] Shi Y (2020). Site-specific electrodeposition enables self-terminating growth of atomically dispersed metal catalysts. Nat. Commun..

[43] Tang J (2019). Advantages of eutectic alloys for creating catalysts in the realm of nanotechnology-enabled metallurgy. Nat. Commun..

[44] Wang Y (2019). Generating defect-rich bismuth for enhancing the rate of nitrogen electroreduction to ammonia. Angew. Chem. Int. Ed..

[45] Dubale AA (2020). High-performance bismuth-doped nickel aerogel electrocatalyst for the methanol oxidation Reaction. Angew. Chem. Int. Ed..

[46] Park J-W, Park C-M (2015). A fundamental understanding of Li insertion/extraction behaviors in SnO and SnO_2_. J. Electrochem. Soc..

[47] Wang J (2019). Linkage effect in the heterogenization of cobalt complexes by doped graphene for electrocatalytic CO_2_ reduction. Angew. Chem. Int. Ed..

[48] Song Y (2020). Dry reforming of methane by stable Ni–Mo nanocatalysts on single-crystalline MgO. Science.

[49] Liu H (2021). Solid–liquid phase transition induced electrocatalytic switching from hydrogen evolution to highly selective CO_2_ reduction. Nat. Catal..

[50] Negishi N, Miyazaki Y, Kato S, Yang Y (2019). Effect of HCO^3−^ concentration in groundwater on TiO_2_ photocatalytic water purification. Appl. Catal. B.

[51] Chernyshova IV, Somasundaran P, Ponnurangam S (2018). On the origin of the elusive first intermediate of CO_2_ electroreduction. Proc. Natl Acad. Sci. USA.

[52] Mączka M, Ptak M, Macalik L (2014). Infrared and Raman studies of phase transitions in metal–organic frameworks of [(CH_3_)_2_NH_2_][M(HCOO)_3_] with M = Zn, Fe. Vib. Spectrosc..

[53] Dunwell M (2017). The central role of bicarbonate in the electrochemical reduction of carbon dioxide on gold. J. Am. Chem. Soc..

[54] Goyal A, Marcandalli G, Mints VA, Koper MTM (2020). Competition between CO_2_ reduction and hydrogen evolution on a gold electrode under well-defined mass transport conditions. J. Am. Chem. Soc..

[55] Zhang M (2021). Engineering a conductive network of atomically thin bismuthene with rich defects enables CO_2_ reduction to formate with industry-compatible current densities and stability. Energy Environ. Sci..

[56] Han N (2018). Ultrathin bismuth nanosheets from in situ topotactic transformation for selective electrocatalytic CO_2_ reduction to formate. Nat. Commun..

[57] Tian J (2021). Bi–Sn oxides for highly selective CO_2_ electroreduction to formate in a wide potential window. ChemSusChem.

[58] Yuan T (2020). Two-dimensional amorphous SnO_x_ from liquid metal: mass production, phase transfer, and electrocatalytic CO_2_ reduction toward formic acid. Nano Lett..

[59] Kresse G, Furthmüller J (1996). Efficient iterative schemes for ab initio total-energy calculations using a plane-wave basis set. Phys. Rev. B.

[60] Blöchl PE (1994). Projector augmented-wave method. Phys. Rev. B.

[61] Perdew JP, Burke K, Ernzerhof M (1996). Generalized gradient approximation made simple. Phys. Rev. Lett..

[62] von Barth U, Hedin L (1972). A local exchange-correlation potential for the spin polarized case. J. Phys. C Solid State Phys..

[63] Grimme S, Antony J, Ehrlich S, Krieg H (2010). A consistent and accurate ab initio parametrization of density functional dispersion correction (DFT-D) for the 94 elements H-Pu. J. Chem. Phys..

[64] Ren B, Li J, Wen G, Ricardez−Sandoval L, Croiset E (2018). First-principles based microkinetic modeling of CO_2_ reduction at the Ni/SDC cathode of a solid oxide electrolysis cell. J. Phys. Chem. C..

[65] Ren B, Wen G, Ricardez–Sandoval L, Croiset E (2021). New mechanistic insights into CO_2_ reduction in solid oxide electrolysis cell through a multi-scale modelling approach. J. Power Sources.

[66] Johnson, R. D. *NIST computational chemistry comparison and benchmark database*. http://srdata.nist.gov/cccbdb (2006).

[67] Tang W, Sanville E, Henkelman G (2009). A grid-based Bader analysis algorithm without lattice bias. J. Phys. Condens. Matter.

[68] Li G (2017). Design of ultralong single-crystal nanowire-based bifunctional electrodes for efficient oxygen and hydrogen evolution in a mild alkaline electrolyte. J. Mater. Chem. A.

