# Supplementary material for: Nano-crumples induced Sn-Bi bimetallic interface pattern with moderate electron bank for highly efficient CO2 electroreduction
Source: Nat Commun. 2022 May 5;13:2486. doi: 10.1038/s41467-022-29861-w (PMC9072316; doi:10.1038/s41467-022-29861-w)
Supplement: Supplementary file 1 — Supplementary Information [file 41467_2022_29861_MOESM1_ESM.pdf]

Supplementary Information

Nano-crumples induced Sn-Bi bimetallic interface pattern with moderate electron bank for highly efficient CO_2 electroreduction

Bohua Ren[1,2,=], Guobin Wen[1,2,=], Rui Gao[2], Dan Luo[1,2], Zhen Zhang[2], Weibin Qiu[1,2], Qianyi Ma[2], Xin Wang[1,3,*], Yi Cui[4], Luis Ricardez–Sandoval[2,*], Aiping Yu[2,*] & Zhongwei Chen[2,*]

[1] Guangdong Provincial Key Laboratory of Nanophotonic Functional Materials and Devices, School of Information and Optoelectronic Science and Engineering & International Academy of Optoelectronics at Zhaoqing, South China Normal University, Guangdong 510631, China.

[2] Department of Chemical Engineering, Waterloo Institute for Nanotechnology, Waterloo Institute for Sustainable Energy, University of Waterloo, 200 University Avenue West, Waterloo, Ontario N2L 3G1, Canada.

[3] South China Academy of Advanced Optoelectronics, South China Normal University, Guangdong 510006, China.

[4] Vacuum Interconnected Nanotech Workstation, Suzhou Institute of Nano-Tech and Nano-Bionics, Chinese Academy of Sciences, Suzhou, 215123 China.

E-mail: wangxin@scnu.edu.cn; laricard@uwaterloo.ca; aipingyu@uwaterloo.ca; zhwchen@uwaterloo.ca

[=] These authors contributed equally to this work.

Inventory of Supplementary Information:

Supplementary Tables 1 to 8

Supplementary Figures 1 to 29

References 1 to 51

1. Supplementary Tables

Supplementary Table 1. Summary of different combinations of Sn and Bi in the unit cell considered in this study.

Unit cells	Cleaved surfaces	Energies (eV)
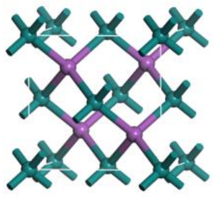	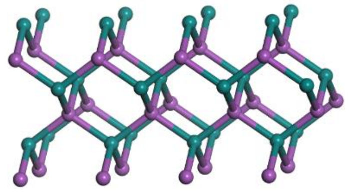	-175.43
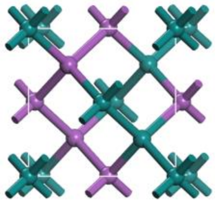	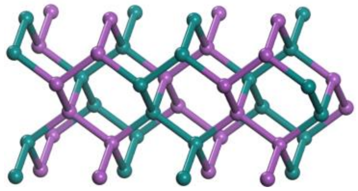	-175.09
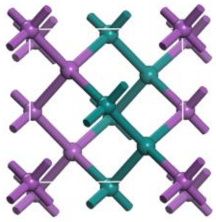	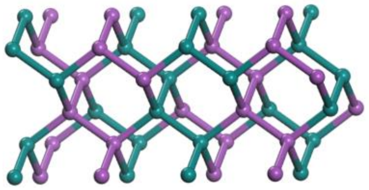	-174.91
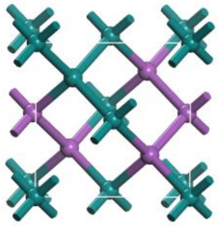	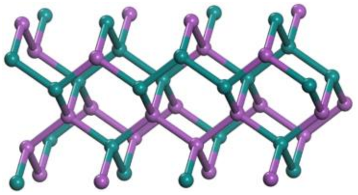	-175.17
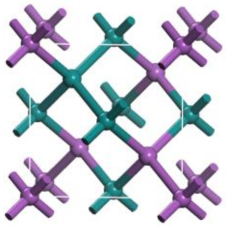	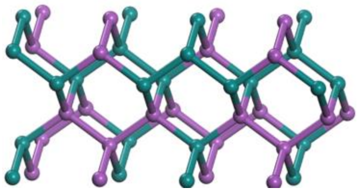	-175.25

Supplementary Table 2. Adsorption energies of intermediates on three surface models.

Intermediates	Adsorption energy (eV)		
	SnBi-Alloy (200) surface	Bi-Sn(200) bimetallic interface	Pure Sn(200) surface
CO_3H	-2.58	-3.64	-2.97
COOH	-1.74	-1.62	-1.75
HCOO	-3.18	-3.09	-2.82

Supplementary Table 3. Formal charges calculated through Bader Charge analysis.

Adsorbates	Atoms	Surface models		
		SnBi-Alloy (200) surface	Bi-Sn(200) bimetallic interface	Pure Sn(200) surface
COOH*	C	1.208621	1.208814	1.20827
	O1	-1.2779	-1.25414	-1.12386
	O2	-1.09254	-1.08668	-1.07926
	Sn[a]	0.297361	0.274557	0.274452
HCOO*	C	1.477862	1.476631	1.504308
	O1[b]	-1.13016	-1.12593	-1.11281
	O2	-1.15342	-1.14918	-1.14602
	Sn[c]	0.513719	0.486418	0.626379

Note: a: binding with C; b: binding with Sn; c: binding with O1 atom.

Supplementary Table 4. Summary of the Sn K-edge fitting results of *in-situ* FT-EXAFS for Sn-Bi interface and Sn-Bi alloy and *ex-situ* FT-EXAFS for the reference.

Sample	Sn K-edge EXAFS fittings of Sn-Bi interface [a]						
	Path	CN	R (Å)	χ (%)	σ^2 ($Å^2$) ×1000	ΔE (eV)	R-factor
Sn reference	Sn-Sn	4.0 ± 0.2	3.00 ± 0.01	-	9.0 ± 0.7	9.5 ± 0.9	0.011
OCV	Sn-Sn	2.1 ± 0.2	3.02 ± 0.02	65.8	9.1 ± 0.8	4.8 ± 0.3	0.009
	Sn-Bi	1.1 ± 0.2	3.10 ± 0.02	34.2	7.3 ± 0.5		
-0.64 V	Sn-Sn	2.2 ± 0.3	3.03 ± 0.02	68.6	6.0 ± 0.5	9.1 ± 0.6	0.016
	Sn-Bi	1.0 ± 0.1	3.10 ± 0.01	31.4	6.3 ± 0.5		
-0.84 V	Sn-Sn	2.3 ± 0.2	3.03 ± 0.02	69.9	6.2 ± 0.4	6.8 ± 0.6	0.010
	Sn-Bi	1.0 ± 0.1	3.10 ± 0.02	30.1	6.1 ± 0.5		
-1.04 V	Sn-Sn	2.6 ± 0.2	3.03 ± 0.01	74.4	5.5 ± 0.4	8.4 ± 0.7	0.008
	Sn-Bi	0.9 ± 0.2	3.11 ± 0.02	25.6	7.2 ± 0.5		
-1.24 V	Sn-Sn	2.7 ± 0.3	3.04 ± 0.02	77.0	5.0 ± 0.4	5.2 ± 0.3	0.012
	Sn-Bi	0.8 ± 0.2	3.11 ± 0.03	23.0	9.1 ± 0.8		
Sample	Sn K-edge EXAFS fittings of Sn-Bi alloy [b]						
	Path	CN	R (Å)	χ (%)	σ^2 ($Å^2$) ×1000	ΔE (eV)	R-factor
OCV	Sn-Sn	1.6 ± 0.2	3.04 ± 0.02	47.4	5.9 ± 0.4	6.4 ± 0.5	0.015
	Sn-Bi	1.8 ± 0.3	3.09 ± 0.01	52.6	9.1 ± 0.7		
-0.64 V	Sn-Sn	1.8 ± 0.1	3.04 ± 0.02	51.1	5.5 ± 0.4	8.9 ± 0.8	0.014
	Sn-Bi	1.7 ± 0.1	3.09 ± 0.02	48.9	7.2 ± 0.7		
-0.84 V	Sn-Sn	1.9 ± 0.2	3.03 ± 0.02	53.9	8.2 ± 0.7	6.0 ± 0.4	0.010
	Sn-Bi	1.6 ± 0.1	3.10 ± 0.01	46.1	7.8 ± 0.5		
-1.04 V	Sn-Sn	2.1 ± 0.3	3.04 ± 0.02	60.1	6.6 ± 0.6	7.5 ± 0.5	0.012
	Sn-Bi	1.4 ± 0.1	3.11 ± 0.02	39.9	9.8 ± 0.6		
-1.24 V	Sn-Sn	2.3 ± 0.3	3.03 ± 0.03	64.1	7.9 ± 0.8	4.6 ± 0.3	0.019
	Sn-Bi	1.3 ± 0.2	3.11 ± 0.02	35.9	8.9 ± 0.7		

Note: CN is the coordination numbers; R is the interatomic distance (the bond length between Sn central atoms and surrounding coordination atoms); χ represents a hetero-atomic intermix of coordination neighbors to the target atom in a selected bond pair; σ^2 is Debye-Waller factor (a measure of thermal and static disorder in absorber-scatterer distances); ΔE is an inner potential correction (the difference between the zero kinetic energy value of the sample and that of the theoretical model); R-factor is used to value the goodness of the fitting.

[a,b] S_0^2 is the amplitude reduction factor. The CN of Sn reference was fixed as the nominal values to obtain S_0^2 (0.91 ± 0.04). Then S_0^2 was fixed in the subsequent fitting. The bond lengths and disorder factors of Sn-Bi and Bi-Sn bonds are constrained to be the same during the simultaneous fitting of Sn K-edge and Bi L_3-edge EXAFS spectra. Data ranges: *k*-range = 2.2 – 10.0 $Å^{-1}$; d*k* =1; R-range = 2.0 – 3.6 Å; Reasonable R-factors ensure the good fitting results.

Supplementary Table 5. Summary of the Bi L_3-edge fitting results of *in-situ* FT-EXAFS for Sn-Bi interface and Sn-Bi alloy and *ex-situ* FT-EXAFS for the reference.

Sample	Bi L_3-edge EXAFS fittings of Sn-Bi interface [c]						
	Path	CN	R (Å)	χ (%)	σ^2 (Å^2) ×1000	ΔE (eV)	R-factor
Bi reference	Bi-Bi	3.0 ± 0.1	3.16 ± 0.02	-	3.2 ± 0.2	5.2 ± 0.4	0.009
OCV	Bi-Bi	1.8 ± 0.2	3.14 ± 0.01	58.7	9.1 ± 0.8	6.9 ± 0.6	0.014
	Bi-Sn	1.2 ± 0.1	3.10 ± 0.02	41.3	7.3 ± 0.5		
-0.64 V	Bi-Bi	1.7 ± 0.3	3.14 ± 0.01	59.3	6.0 ± 0.5	7.2 ± 0.6	0.016
	Bi-Sn	1.2 ± 0.1	3.10 ± 0.01	40.7	6.3 ± 0.5		
-0.84 V	Bi-Bi	1.7 ± 0.2	3.16 ± 0.01	61.1	6.2 ± 0.5	8.0 ± 0.7	0.014
	Bi-Sn	1.1 ± 0.2	3.10 ± 0.02	38.9	6.1 ± 0.5		
-1.04 V	Bi-Bi	1.9 ± 0.1	3.16 ± 0.02	62.9	8.5 ± 0.7	6.6 ± 0.6	0.010
	Bi-Sn	1.1 ± 0.1	3.11 ± 0.02	37.1	7.2 ± 0.5		
-1.24 V	Bi-Bi	1.9 ± 0.2	3.17 ± 0.02	64.4	5.0 ± 0.5	4.9 ± 0.4	0.016
	Bi-Sn	1.1 ± 0.1	3.11 ± 0.03	35.6	9.1 ± 0.8		
Sample	Bi L_3-edge EXAFS fittings of Sn-Bi alloy [d]						
	Path	CN	R (Å)	χ (%)	σ^2 (Å^2) ×1000	ΔE (eV)	R-factor
OCV	Bi-Bi	1.5 ± 0.2	3.13 ± 0.01	47.4	5.8 ± 0.4	9.2 ± 0.6	0.012
	Bi-Sn	1.3 ± 0.2	3.09 ± 0.01	52.6	9.1 ± 0.7		
-0.64 V	Bi-Bi	1.5 ± 0.1	3.14 ± 0.02	51.1	5.4 ± 0.4	7.7 ± 0.6	0.011
	Bi-Sn	1.2 ± 0.1	3.09 ± 0.02	48.9	7.2 ± 0.7		
-0.84 V	Bi-Bi	1.6 ± 0.2	3.15 ± 0.01	53.9	8.5 ± 0.7	4.8 ± 0.7	0.015
	Bi-Sn	1.2 ± 0.2	3.10 ± 0.01	46.1	7.8 ± 0.5		
-1.04 V	Bi-Bi	1.6 ± 0.2	3.14 ± 0.02	60.1	6.3 ± 0.5	4.2 ± 0.6	0.011
	Bi-Sn	1.3 ± 0.3	3.11 ± 0.02	39.9	9.8 ± 0.4		
-1.24 V	Bi-Bi	1.6 ± 0.1	3.16 ± 0.02	64.1	8.6 ± 0.9	7.9 ± 0.4	0.014
	Bi-Sn	1.2 ± 0.2	3.11 ± 0.02	35.9	8.9 ± 0.7		

[c,d] The CN of Bi reference was fixed as the nominal values to obtain S_0^2 (0.94 ±0.03). Then S_0^2 was fixed in the subsequent fitting. Data ranges: *k*-range = 1.0 – 9.0 Å^{-1}; d*k* =1; R-range = 2.0 – 3.5.

Supplementary Table 6. Summary of the performances of Sn-based and Bi-based electrocatalysts for electrochemical CO_2RR in H-type cell.

Electrocatalysts	Electrolytes	Potentials (V vs. RHE)	FE_{HCOOH} (%)	J_{HCOOH} (mA/cm^2)	References
Crumple induced Sn-Bi interface	0.5 M $KHCO_3$	-0.84	96.4	39.5	This work
		-1.04	92.1	103.1	This work
Bi-Sn Aerogel	0.1 M $KHCO_3$	-1.0	93.9	9.3	1
Eutectic Bi-Sn	0.1 M $KHCO_3$	-1.1	78	10.7	2
Bi-Sn/CF	0.5 M $KHCO_3$	-1.14	94	55	3
Bi-Doped Amorphous SnO_x Nanoshells	0.5 M $NaHCO_3$	-0.88	95.8	20.9	4
		-0.67	91	4	4
BiO_x-decorated SnO_x nanoflake	0.5 M $KHCO_3$	-1.37	90.8	40.5	5
Bi-MOF	0.1 M $KHCO_3$	-0.9	92	4	6
SnO_2 quantum wires	0.1 M $KHCO_3$	-1.16	87.3	13.7	7
Ultrathin Bi nanosheet with vacancies	0.1 M $KHCO_3$	-0.8	97	3.8	8
Hierarchical Mesoporous SnO_2 Nanosheets	0.5 M $NaHCO_3$	-1.0	87	43.5	9
ultra-small SnO nanoparticles	0.5 M $KHCO_3$	-0.86	75	19.5	10
SnO_2 nanoparticles-decorated hollow carbon	0.1 M $KHCO_3$	-0.9	54.2	4	11

BiO_x/C	0.5 M $NaHCO_3$/0.5 M $NaClO_4$	-1.1	93	16	[12]
Bi_2O_3-nitrogen-doped graphene quantum dots	0.5 M $KHCO_3$	-0.9	98	17.5	[13]
Graphene confined Sn quantum sheets	0.1 M $NaHCO_3$	-1.14	89	19	[14]
Sn nanoparticles mixed with graphene	0.1 M $NaHCO_3$	-1.14	60	5.4	[14]
SnO_2 nanocubes	0.5 M $KHCO_3$	-1.0	71	8	[15]
SnO_2 nanoflakes	0.5 M $KHCO_3$	-1.0	83	11	[15]
Bi_2O_3-5 h	0.5 M $KHCO_3$	-0.9	91	8	[16]
SnO_x porous nanowires	0.1 M $KHCO_3$	-1.0	80	7.4	[17]
SnO_x Nanosheets @MWCNTs	0.5 M $KHCO_3$	-1.25	77	9.2	[18]
SnO_2 nanocluster	0.5 M $NaHCO_3$	-0.92	73	16.3	[19]
$SnO_x(OH)_y$	0.1 M KCl	-1.16	82	13	[20]
Sn/SnO_x porous hollow fiber	0.1 M $KHCO_3$	-0.93	82.1	22.9	[21]
Ag-Sn bimetallic core/SnO_x shell	0.5 M $NaHCO_3$	-0.8	~80	16	[22]
Cu/SnO_2 Core/Shell	0.5 M $KHCO_3$	-0.7	93	~10	[23]
Cu NPs/hollow SnO_2 Janus structure	0.1 M $KHCO_3$	-1.1	68	10	[24]

CuO/ hollow SnO_2 heterostructure	0.1 M $KHCO_3$	-1.0	71.5	12	[24]
$Cu_{41}Sn_{11}$@SnO_2 core–shell	0.1 M $KHCO_3$	-1.0	70	10.8	[24]
Bi_2O_3 nanosheets @conductive multiple channel carbon matrix	0.1 M $KHCO_3$	-1.26	93.8	15	[25]
Bi nanotubes	0.5 M $KHCO_3$	-1.0	97	25	[26]
Ultrathin bismuth nanosheets	0.5 M $NaHCO_3$	-0.84	96	13	[27]
Porous conductive network of bismuthene	0.5 M $KHCO_3$	-0.97	95	67	[28]

Supplementary Table 7. Summary of the performances of Sn-based and Bi-based electrocatalysts for electrochemical CO_2RR in flow-type cells.

Electrocatalysts	Electrolytes	Potentials (V vs. RHE)	FE_{HCOOH} (%)	$PCD_{formate}$ (mA/cm^2)	Stability (h)		References
Crumple induced Sn-Bi interface	0.5 M $KHCO_3$	-1.04	90	171	116 (-1.04V, total CD[a] of 188 mA/cm^2)	50	This work
		-1.24	72	230		37	This work
Sn-Bi/SnO_2	1.0 M $KHCO_3$ and KOH (pH=8)[c]	-1.20	95	114	2400(pH=11, ~100 mA/cm^2)	49	[29]
Defective $Bi_2O_2CO_3$ nanosheet	0.5 M $KHCO_3$	-1.20	-	50 (total CD)	-	-	[30]
	1.0 M KOH (pH=14)	-1.15	91	550	< 24 (~ 200 mA/cm^2)	48	[30]
SnO_2 nanosheet	1.0 M KOH	-1.13	92	450	-	49	[31]
Atomically thin bismuthene (Bi-ene)	0.5 M $KHCO_3$	-1.20	-	75 (total CD[a])	1	-	[32]
	1.0 M KOH	-0.75	~100	~200	1	63	[32]
Leafy bismuth nanosheets	1.0 M $KHCO_3$	-1.20	92	138	10	55	[33]
	1.0 M KOH	-0.60	91	273	10	72.5	[33]
Ultrathin two-dimensional Bi	2.0 M KOH (pH=14.2)	-0.68	85	183	-	56	[34]
Oxide derived Bi	1.0 M $KHCO_3$	-1.05	86	125	100 (~30 mA/cm^2)	47	[35]
nBuLi-Bi	1.0 M $KHCO_3$	-1.05	95	475	100 (~30 mA/cm^2)	53	[35]
Sn-Cu/SnO_x core/shell	1.0 M KOH	-0.70	87	353	40(~243 mA/cm^2)	57	[36]
Sn	0.45 M $KHCO_3$ +	-1.50	70	105	-	32	[37]

	0.5 M KCl						
Ultra-small SnO_2 nanoparticles	1.0 M $KHCO_3$	-1.21	64	93	-	33	[38]
	1.0 M KOH	-0.95	46	88	-	27	[38]

[a] CD represents current density.

[b] The cathodic energetic efficiency (EE_{ca}) for CO_2RR to formate was calculated using the following equation[39, 40]:

$$EE_{ca} = \frac{E_{eq,cell} \cdot FE}{E_{eq,cell} + \eta_{cathode}}$$

Where, $E_{eq,cell}$ is the thermodynamic equilibrium potential between anode and cathode reactions, which is $E_{eq,cell} = 1.23 + (-E_{eq,formate})$; 1.23 V is the thermodynamic equilibrium potential for anode OER reaction; $E_{eq,formate}$ is the thermodynamic equilibrium potential for cathode reaction to formate (vs RHE). $E_{eq,formate} = -0.03$ V; $\eta_{cathode}$ is the cathode overpotential, which is defined as the applied cathode potential (vs. RHE) minus the thermodynamic equilibrium potential.

Most reports did not collect the full-cell potential when doing CO_2RR measurements in a three-electrode configuration, so the applied potential at the anode was assumed to be 1.23 V[29]. Note that the actual full-cell EE might be smaller than the cathodic energy efficiency calculated here since the anodic side should also show an overpotential for the oxygen revolution reaction (OER).

[c] It is also important to note that the electrolyte in the flow cell acts a crucial role. To do a fair comparison, the huge impact of pH value[41-45] should be considered. We adopted neutral electrolyte (CO_2 saturated 0.5 M $KHCO_3$) in this study for the flow cell performance test rather than the strong alkaline KOH solution. This is because even though an alkaline solution (e.g. KOH) could increase the electrolyte conductivity (σ_{lyte} is 194 $mS \cdot cm^{-1}$ of 1 M KOH solution) and decrease the overpotential[41, 42], some critical issues remain to be addressed, i.e. the input CO_2 would be severely lost to bicarbonate and carbonate (~ 25% carbon losses)[43-45]. Another reason to avoid the use of KOH solution is that the accumulation of carbonate salt from the reaction between CO_2 and OH^- at the electrode-electrolyte interface will block the pores of the GDL, which causes a gradual decrease in electron conductivity (voltage losses) and inefficient mass transportation[46].

Supplementary Table 8. Double-layer capacitance and the corresponding ECSA.

Sample	Capacitance	ECSA
Sn-Bi interface	6.72 mF/cm^2	112.0
Sn-Bi alloy	4.88 mF/cm^2	81.3
Bi ED	4.35 mF/cm^2	72.5
SnO_x	3.61 mF/cm^2	60.2
Sn ED	4.49 mF/cm^2	74.8
Carbon fabrics	1.77 mF/cm^2	29.5
Reference metal foil[47]	0.06 mF/cm^2	1.0

2. Supplementary Figures

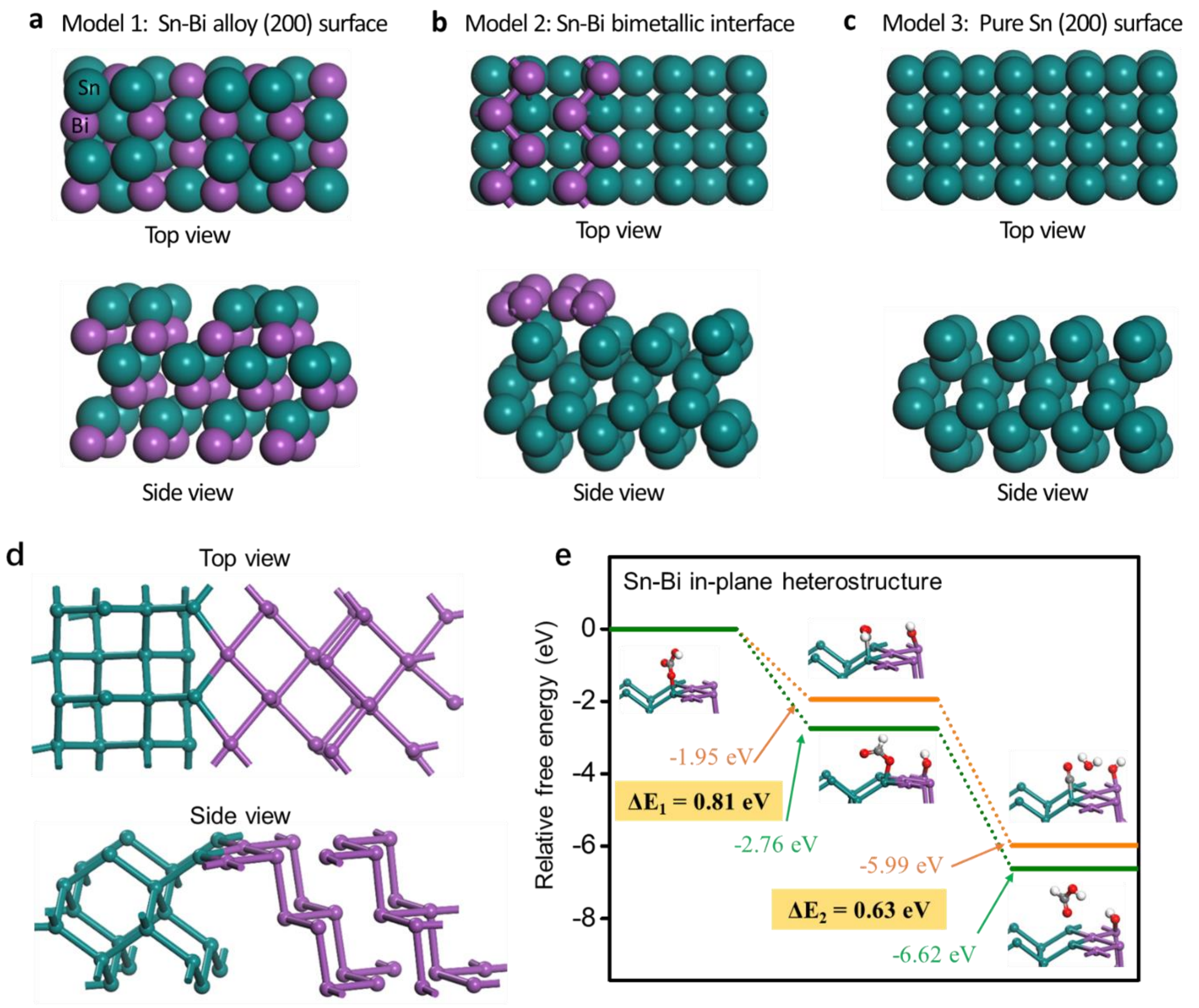

Supplementary Fig. 1 Top view and side view of surface models built for DFT calculations: (a) Sn-Bi alloy (200) surface model. (b) Sn-Bi bimetallic interface model. (c) Pure Sn (200) surface model. (d) Top view and side view of Sn-Bi in-plane heterostructure. (e) CO_2 reduction activity of the in-plane Sn-Bi heterostructure.

We also studied CO_2 reduction activity on the in-plane Sn-Bi heterostructure. The free energy difference for the intermediates HCOO* and COOH* via the first proton-electron transfer reaction is higher (0.81 eV) compared with that of the used Sn-Bi interface model (0.73 eV) and Sn-Bi alloy surface (0.56 eV). For the second proton-electron transfer reaction step towards HCOOH* or CO* formation, the free energy difference between two competing products is almost the same (0.63 eV) compared to that predicted with the Sn-Bi interface model (0.64 eV) and higher than that observed on Sn-Bi alloy surface (0.44 eV). These results indicate that the Sn-Bi in-plane

heterostructure model has slightly higher activity in the HCOO* formation and almost the same activity of HCOOH* formation compared to the Sn-Bi interface model considered in this study. Also, it was confirmed that both Sn-Bi interface models (current interface model and the in-plane model) exhibit higher activity towards formate formation than the Sn-Bi bulk alloy model. These results are consistent with our conclusion, i.e., Sn-Bi bimetallic interface has higher activity than that of the Sn-Bi bulk alloy.

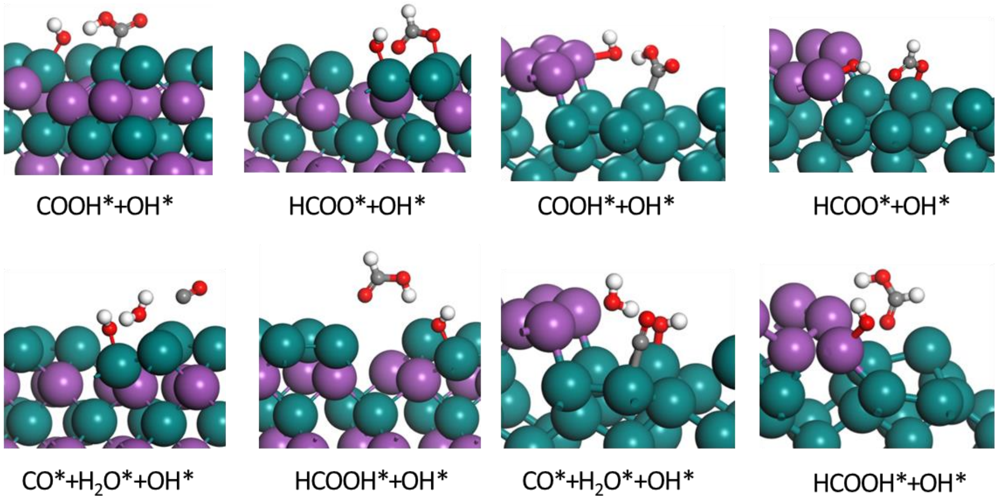

Supplementary Fig. 2 Optimized structures of the involved intermediates of the two competing reaction pathways considered in this study adsorbed on the surface models of Sn-Bi alloy (200) surface model and Sn-Bi bimetallic interface model.

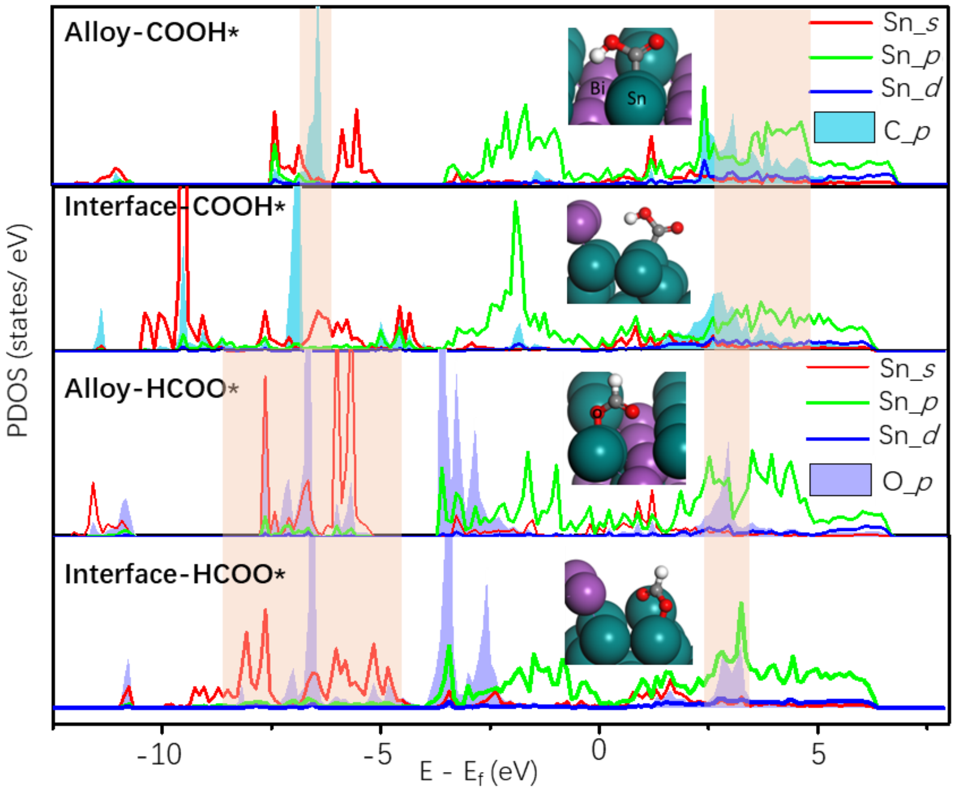

Supplementary Fig. 3 PDOS of *s*, *p*, and *d* orbitals of Sn atom and *p* orbitals of C/O atom on Sn-Bi alloy (200) and Bi-Sn (200) surfaces with adsorbed COOH*/HCOO*. The inset pictures are the corresponding adsorption configurations of involved intermediates.

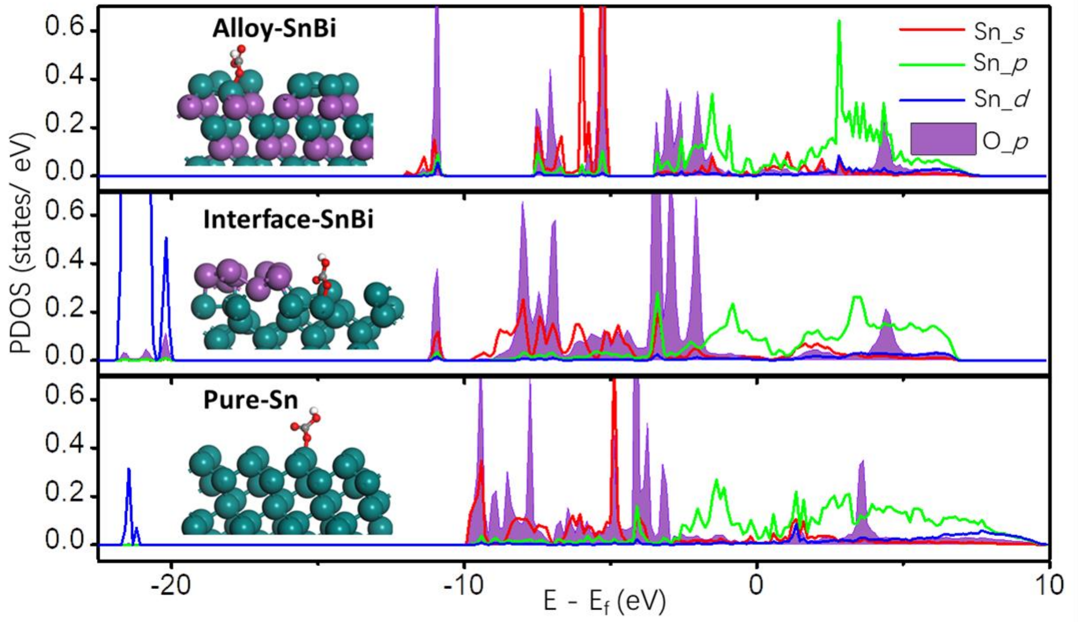

Supplementary Fig. 4 PDOS of *s*, *p*, and *d* orbitals of Sn atom and *p* orbitals of O atom on Sn-Bi alloy (200), Bi-Sn (200) interface and pure Sn (200) surfaces with adsorbed CO_3H*. The inset pictures are the corresponding adsorption configurations of involved intermediates.

To explain the intrinsic mechanism of binding strength sequences of CO_3H*, COOH* and HCOO*, we performed additional DFT calculations to depict the PDOS of *s*, *p*, and *d* orbitals of Sn atom (binding with the O atom of CO_3H*) and *p* orbitals of O atom on Sn-Bi alloy (200), Bi-Sn (200) interface and pure Sn (200) surfaces with adsorbed CO_3H*. The result is shown in Supplementary Fig. 4.

A detailed explanation on the adsorption energies for each of these species and their potential sequences is provided next:

1. CO_3H*

(a) There are more overlapping areas of *p*-*p* and *p*-*s* orbitals between O-2*p* and Sn-5*p*/Sn-5*s* for CO_3H* found on Sn-Bi interface compared with those of alloy and pure Sn. Additionally, overlapping areas of *p*-*d* orbitals at around -20 eV ($E-E_f$) between O-2*p* and Sn-4*d* for CO_3H* were observed on Sn-Bi interface only. These results indicate that the adsorption of CO_3H on Sn-Bi interface is the strongest compared to those of pure Sn and Sn-Bi bulk alloy.

(b) A narrow bandwidth of metal *d* orbital enables a strong substrate–adsorbate coupling. This is mostly because the coupling leads to a larger energy split of hybrid orbitals when it is

incorporated with the adsorbates, pushing the antibonding level closer to Fermi level and reducing the energy barrier of electron transport[48-50]. The bandwidth of Sn-4*d* orbital follows the sequence of Bi-Sn(200)<Pure Sn(200)<SnBi-alloy, which is another indication that the CO_3H adsorption follows the sequence: Bi-Sn(200) >Pure Sn(200)>SnBi-alloy.

2. HCOO*

(a) In contrast to CO_3H*, there are no overlapping areas of *p-d* orbitals for HCOO* between O-2*p* and local narrow peak of Sn-4*d* (around -20 eV ($E-E_f$)) on any surface model (Supplementary Fig. 3). Therefore, the adsorption mechanism of this species is different from that observed in CO_3H*.

(b) Moreover, consistent with our previous finding[3], the more electronegative O atoms tend to show stronger binding with Sn atoms that are more electronically depleted due to the addition of Bi, thus boosting the adsorption energy of the HCOO* intermediate. Therefore, the adsorption strength of HCOO* shows positive dependence on the electronic depletion of Sn metal. Consequently, they tend to follow the sequence: SnBi-alloy>Bi-Sn(200)>Pure Sn(200).

3. COOH*

(a) In contrast to the observation made for CO_3H* and HCOO*, there is π back-donation between Sn-C interaction for COOH adsorption. As stated in the manuscript, the adsorption of COOH is the weakest observed on the Bi-Sn(200) interface.

(b) The adsorption energies of COOH on pure Sn(200) and SnBi-alloy are almost the same (-1.75 and -1.74 eV, respectively). The difference is probably due to the intrinsic bandwidth property of the Sn-4*d* orbital which results in the adsorption sequence for CO_3H*: Pure Sn(200)>SnBi-alloy.

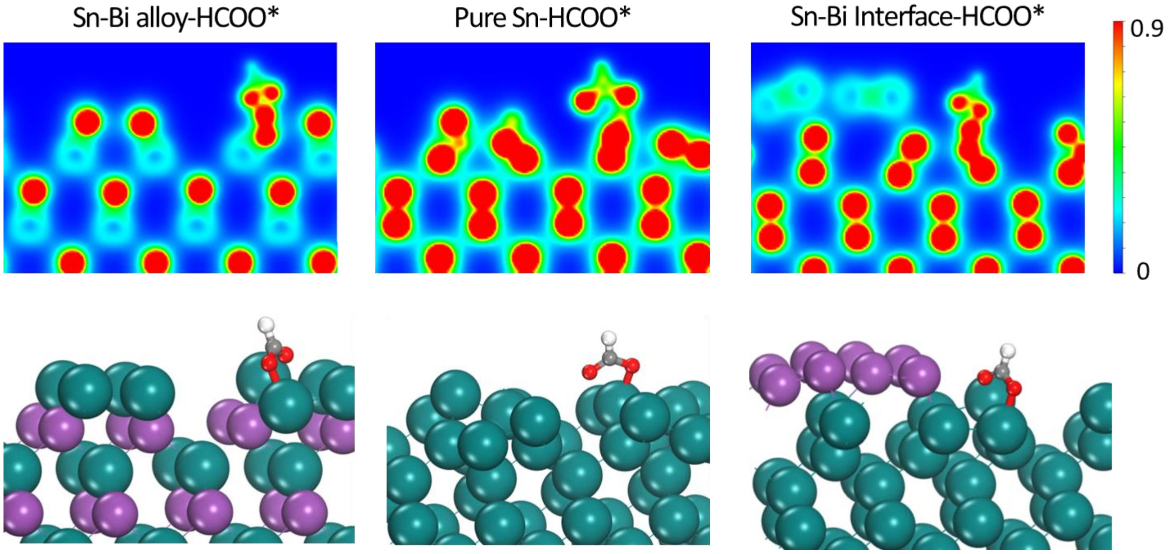

Supplementary Fig. 5 Volume slices of calculated charge densities and corresponding optimized configurations for the three models with HCOO adsorbate.

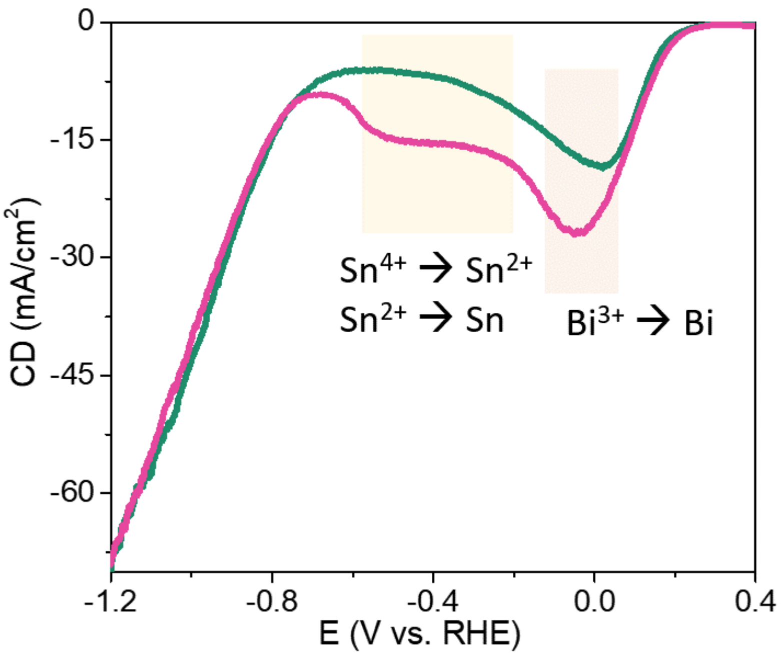

Supplementary Fig. 6 Linear sweep voltammetry curves for Sn and Bi precursors.

The standard reduction potentials at 298.15 K and a pressure of 1 atm of reduction reactions of Sn^{2+} → Sn, Sn^{4+} → Sn^{2+}, and Bi^{3+} → Bi are -0.138 V, 0.151 V, and 0.308 V, respectively. Through conversion to potentials vs RHE, these potentials are -0.56 V, -0.27 V, and -0.12 V, respectively. Supplementary Fig. 5 shows these electrodeposition peaks. In the following synthesis step of constant potential electrodeposition (CP-ED), the potential was set at -0.5 V vs RHE, which was chosen to make both electrodeposition and CO2RR occur at this potential.

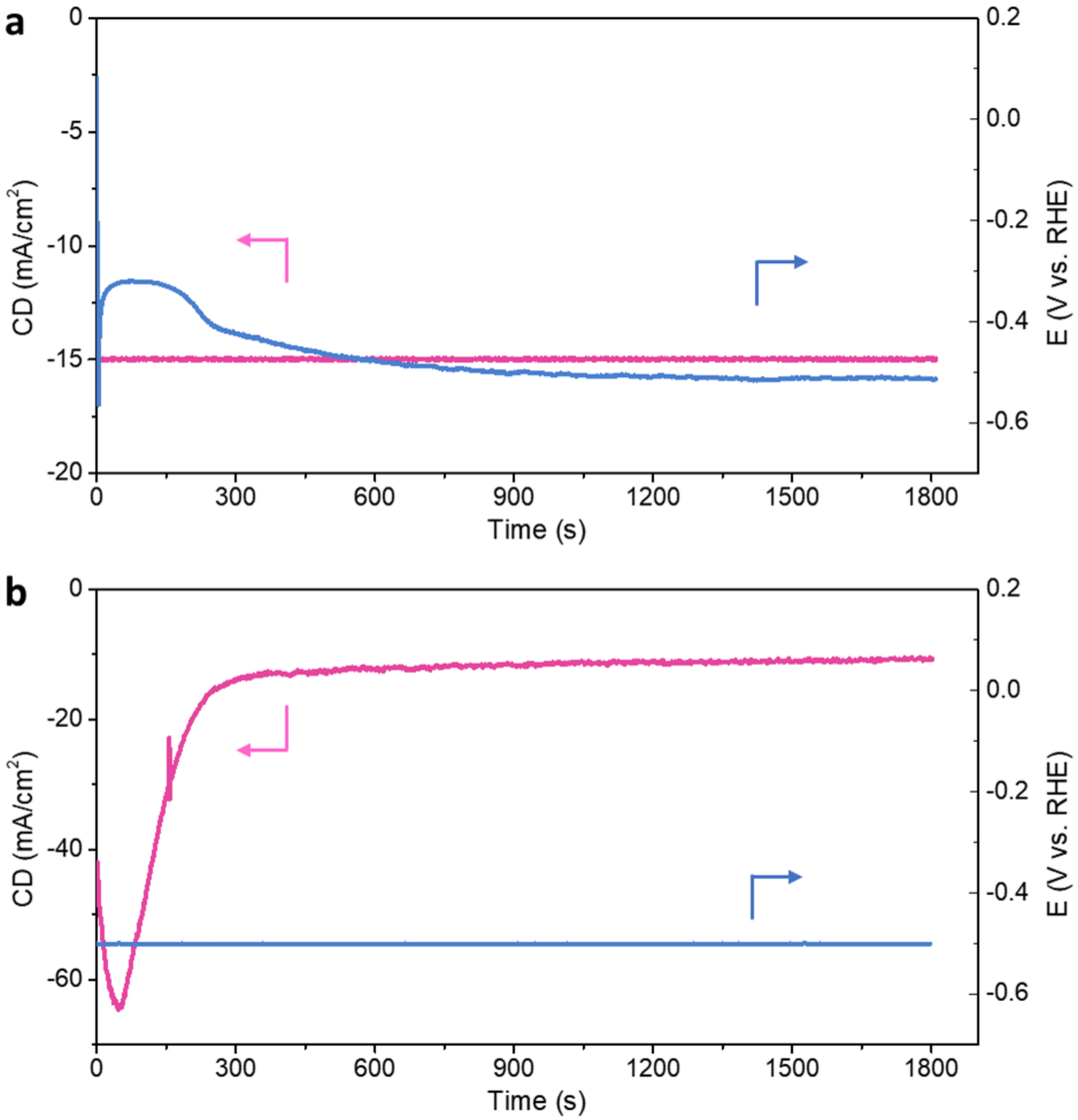

Supplementary Fig. 7 (a) Constant current electrodeposition (CC-ED) at -15 mA/cm^2 for synthesis of crumpled SnBi electrode. (b) Constant potential electrodeposition (CP-ED) at -0.5 V vs. RHE for the reference.

CC-ED provides more stable deposition speed compared with CP-ED, leading to the morphologies of samples by CC-ED are more uniform in Supplementary Fig. 7.

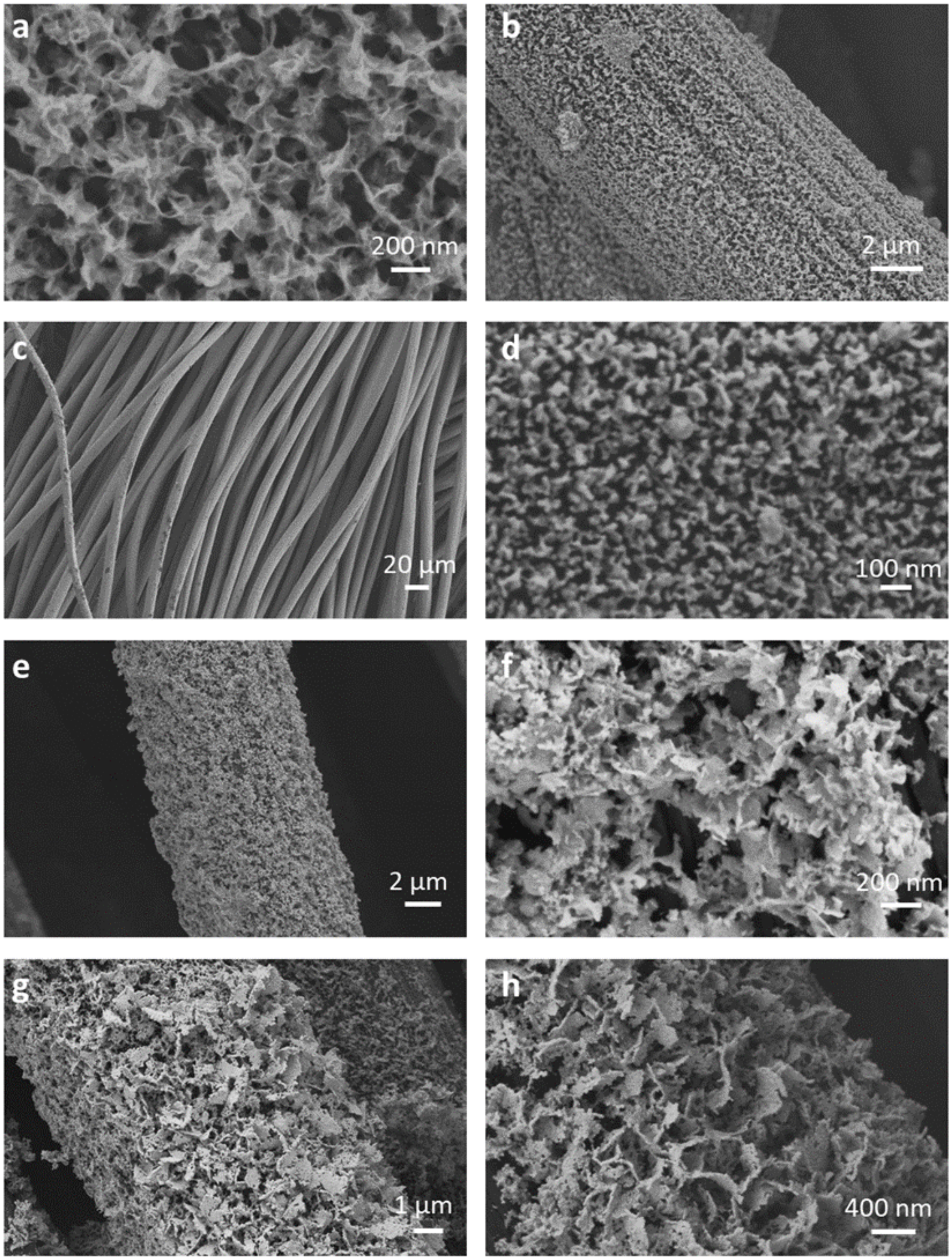

Supplementary Fig. 8 SEM images of Sn-Bi on CF with different ED parameters. (a-c) CC-ED at -15 mA/cm^2 under different magnificence. CP-ED at (d-e) -0.5 V and (f-h) -0.8 V vs. RHE.

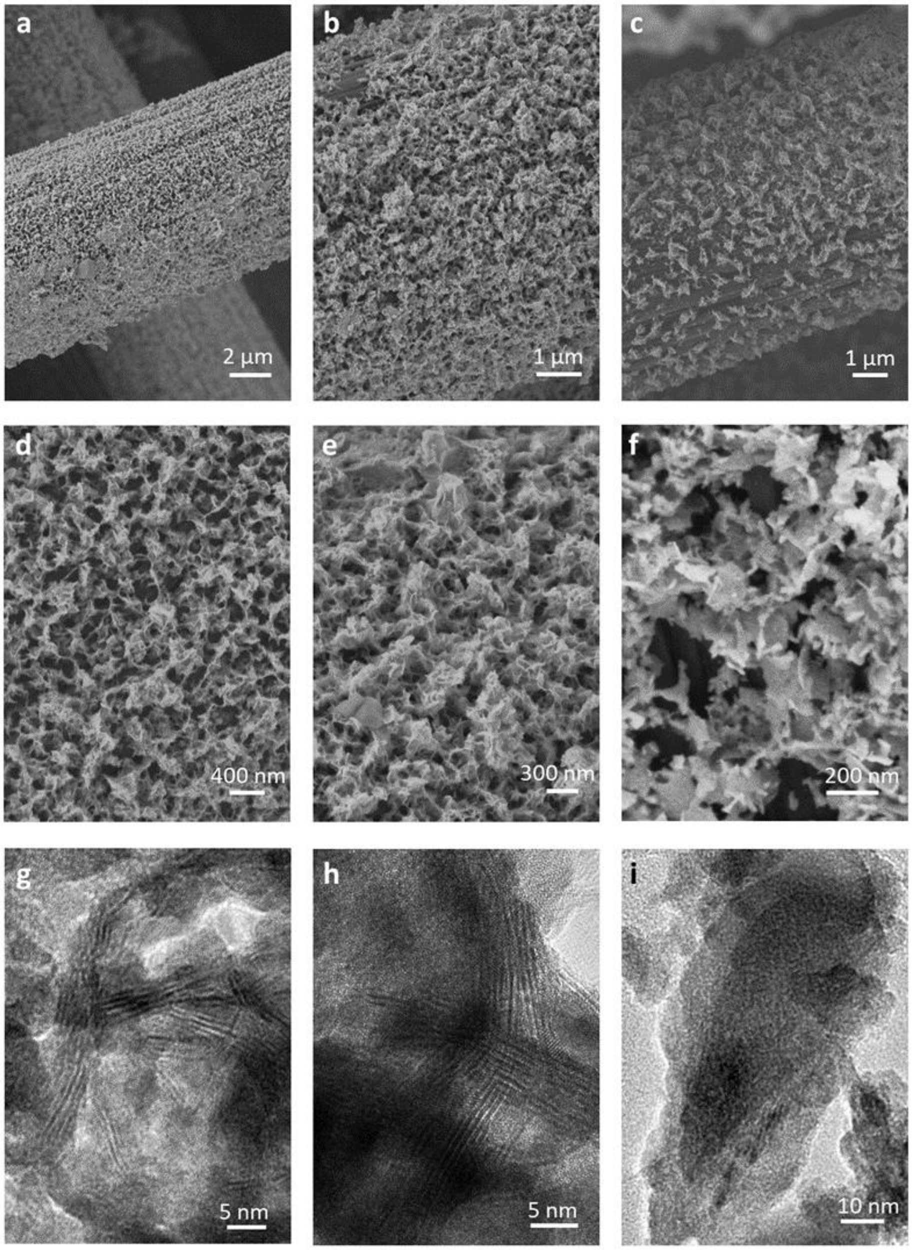

Supplementary Fig. 9 Different density of crumples of different times of CC-ED and flow speed of CO_2 during in-situ ED and evolution. (a, d) SEM images and (g) TEM image of local sites for the samples at -15 mA/cm^2 for 60 mins with CO_2 flow rate of 20 sccm. (b, e) SEM images and (h) TEM image of local sites for the samples at -15 mA/cm^2 for 30 mins with CO_2 flow rate of 40 sccm. (c, f) SEM images and (i) TEM image of local sites for the samples at -15 mA/cm^2 for 30 mins with Ar flow rate of 20 sccm.

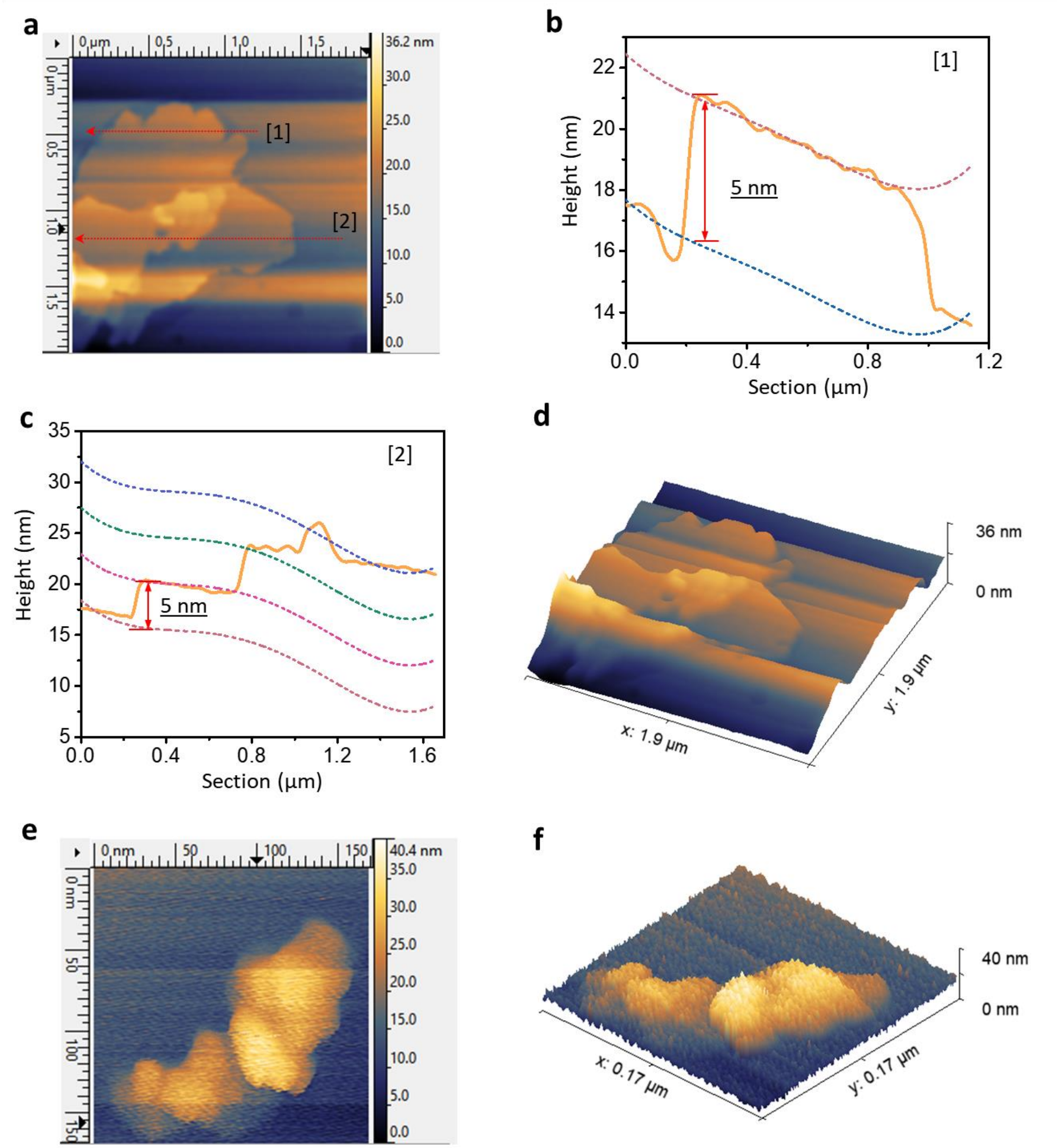

Supplementary Fig. 10 (a) AFM image of flower structures, the corresponding height profile of (b) line 1 and (c) line 2, and (d) three-dimensional AFM image. (e) AFM image in nanometer scale and (f) corresponding three-dimensional AFM image.

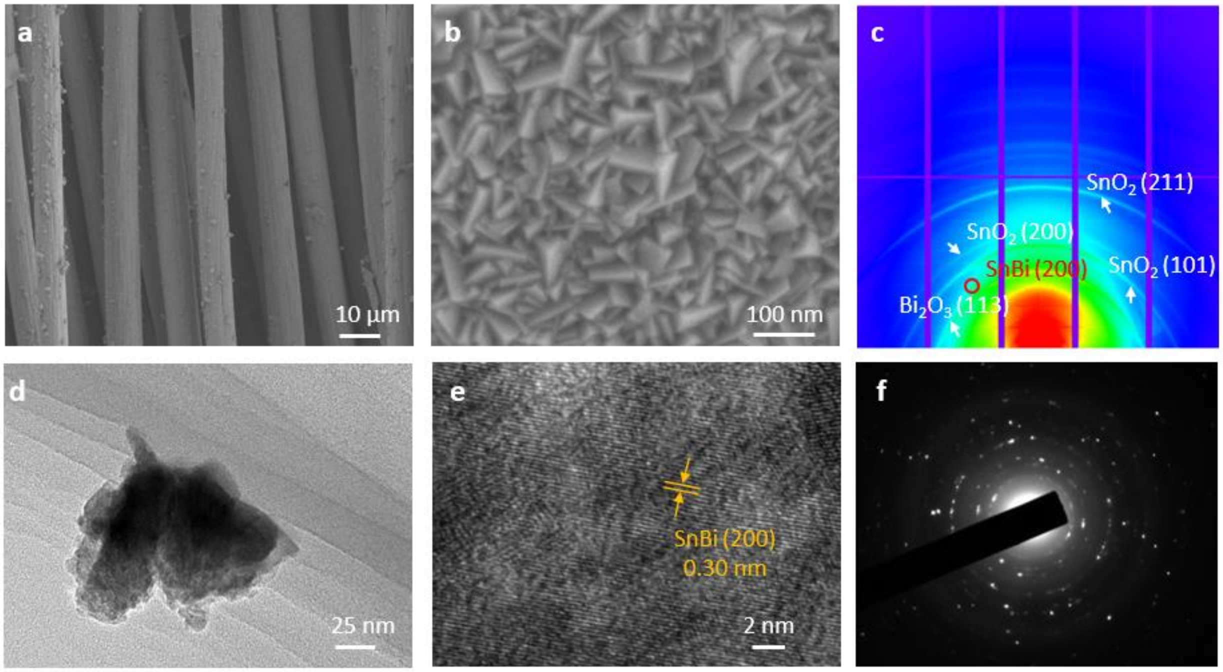

Supplementary Fig. 11 Characterisations of Sn-Bi alloy. (a-b) SEM images. (c) 2D synchrotron GIXD pattern. (d-e) TEM image. (f) selected area electron diffraction (SAED) pattern.

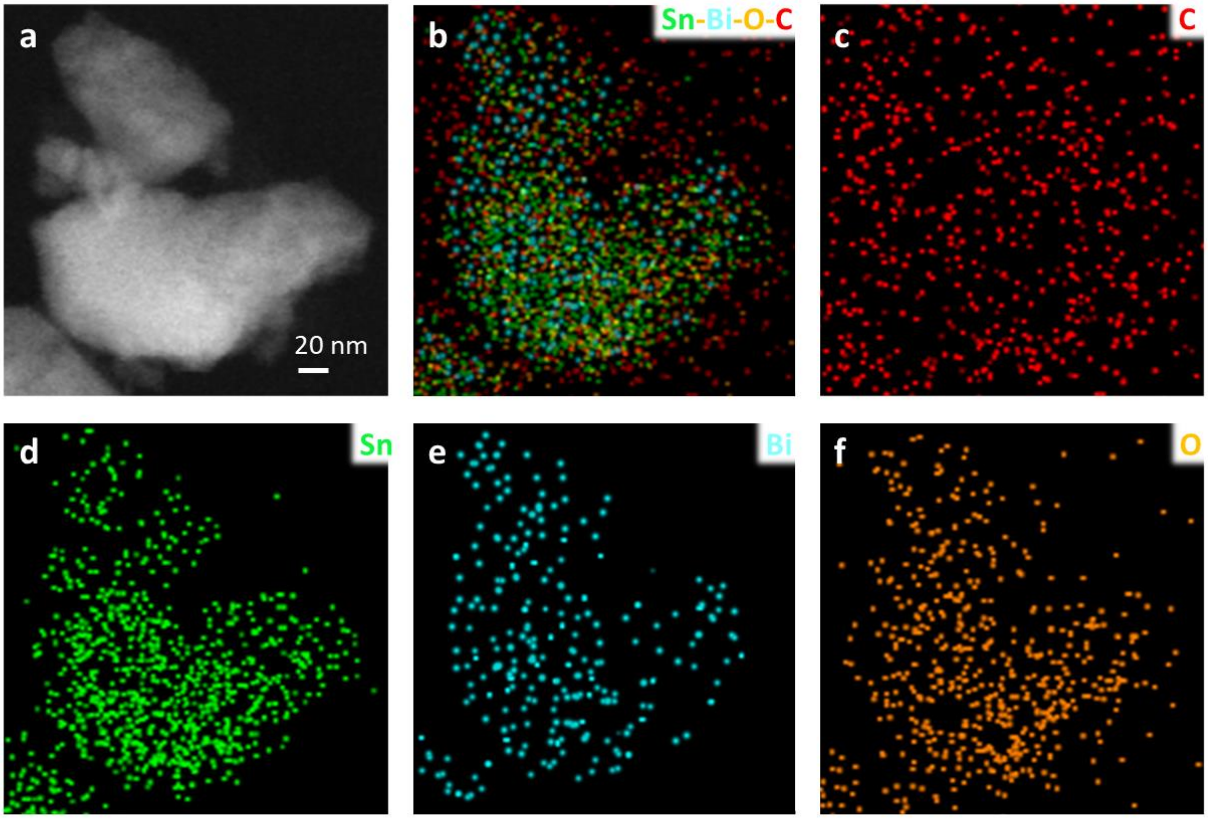

Supplementary Fig. 12 (a) HAADF-STEM image of Sn-Bi alloy and (b-f) corresponding EDS element mapping.

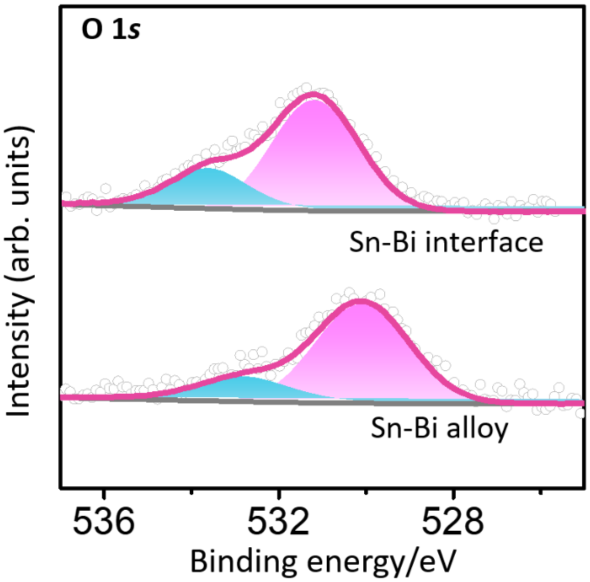

Supplementary Fig. 13 XPS spectra of O1*s* spectrum for Sn-Bi interface and alloy.

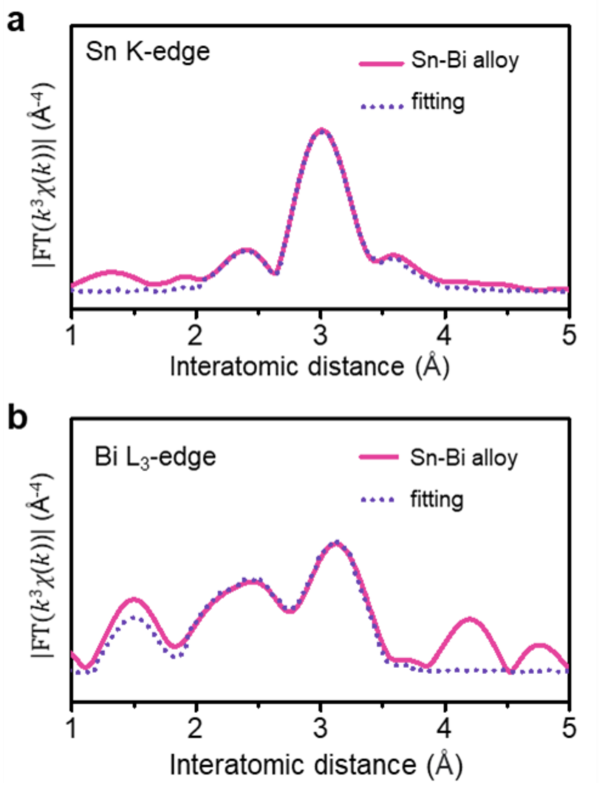

Supplementary Fig. 14 a-b Representative fitting of the EXAFS spectra to the R space of Sn K-edge and Bi L_3-edge of Sn-Bi alloy.

The Sn-Sn (effective bond length: R_{eff} = 3.02 Å) and Sn-Bi paths (R_{eff} = 3.06 Å) were included in the fit which were determined from the CIF files imported from DFT models of pure Sn and Sn-Bi alloy.

The Bi-Bi (R_{eff} = 3.10 Å) and Bi-Sn paths (R_{eff} = 3.06 Å) were included in the fit which were determined from the CIF files imported from DFT models of pure Bi and Sn-Bi alloy.

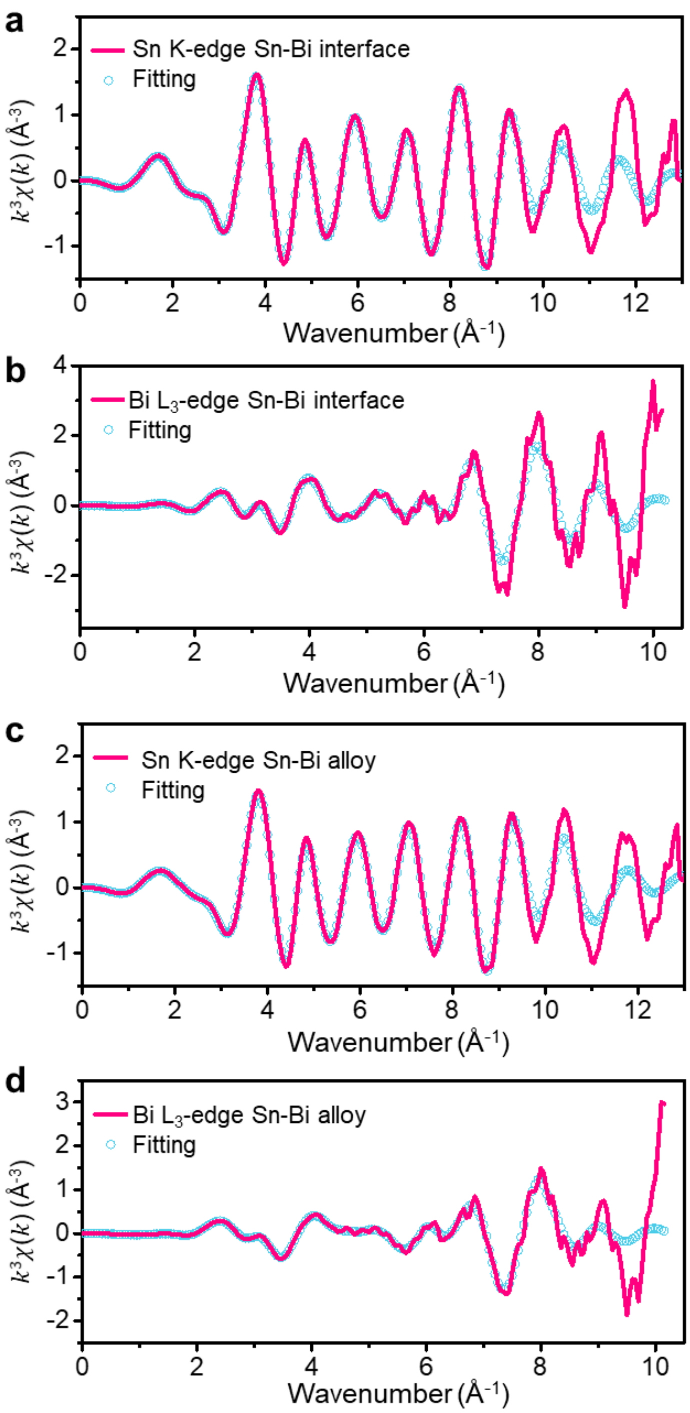

Supplementary Fig. 15 Fitting results of the EXAFS spectra to k space of (a) Sn K-edge and (b) Bi L_3-edge of Sn-Bi interface, (c) Sn K-edge and (d) Bi L_3-edge of Sn-Bi alloy.

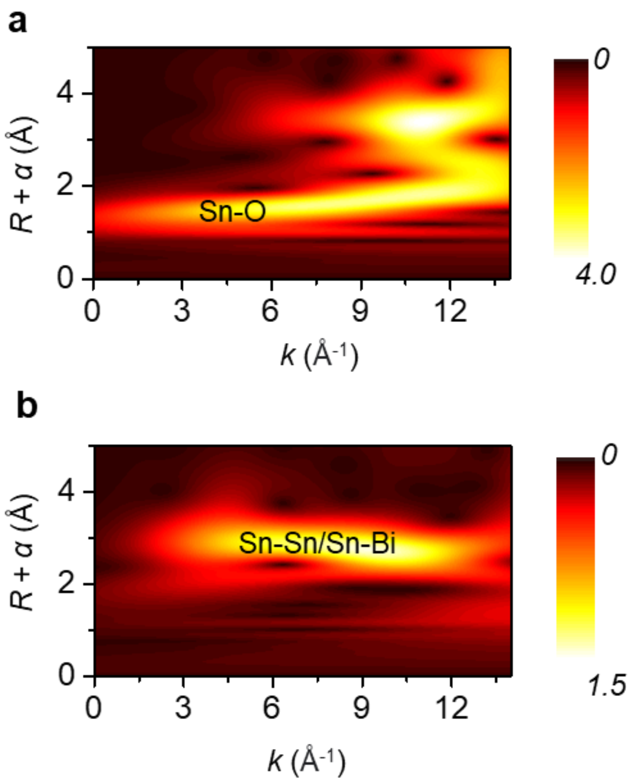

Supplementary Fig. 16 Wavelet transforms of k^3-weighted EXAFS for Sn K-edge in SnO_2 reference and Sn-Bi bimetallic interface materials. The "α" in the Y-axis labels represents a phase shift (ΔR) associated with the scattering event.

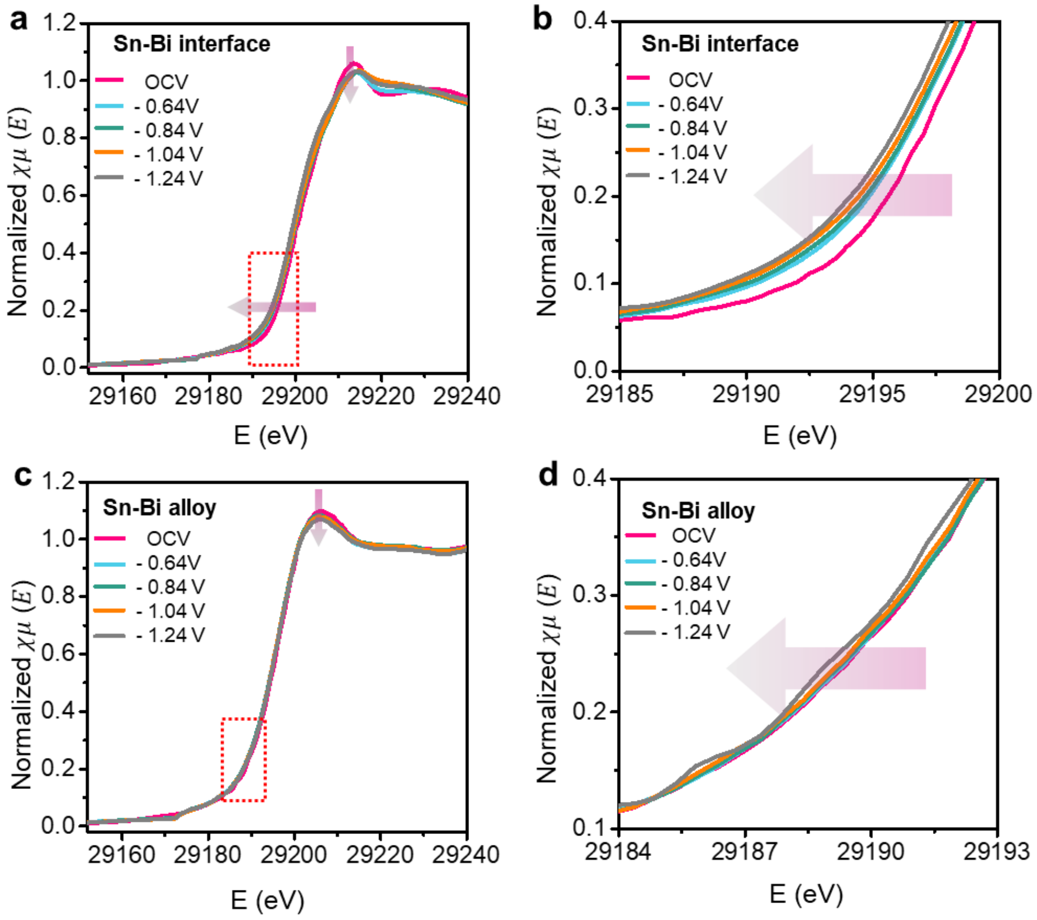

Supplementary Fig. 17. **a, c** *In-situ* XANES spectra of Sn K-edge of Sn-Bi interface and alloy samples respectively, at different applied potentials during CO_2RR. **b, d** The enlarged spectra of the red dashed rectangular area in **a, c** respectively**.**

In general, both the absorption edge and white line peak of Sn K-edge of Sn-Bi interface and alloy show a little shift with applied potentials. The slight lowering of the white line peak intensity and lower-energy shift of absorption edge when decreasing the potentials indicate the slight reduction of Sn under operating conditions[3, 51]. Considering that an increase in Sn-Sn coordination numbers is paralleled by a decrease in Sn-Bi coordination numbers, it is likely that further dealloying of SnBi phase takes place (i.e., further segregation of Sn and Bi).

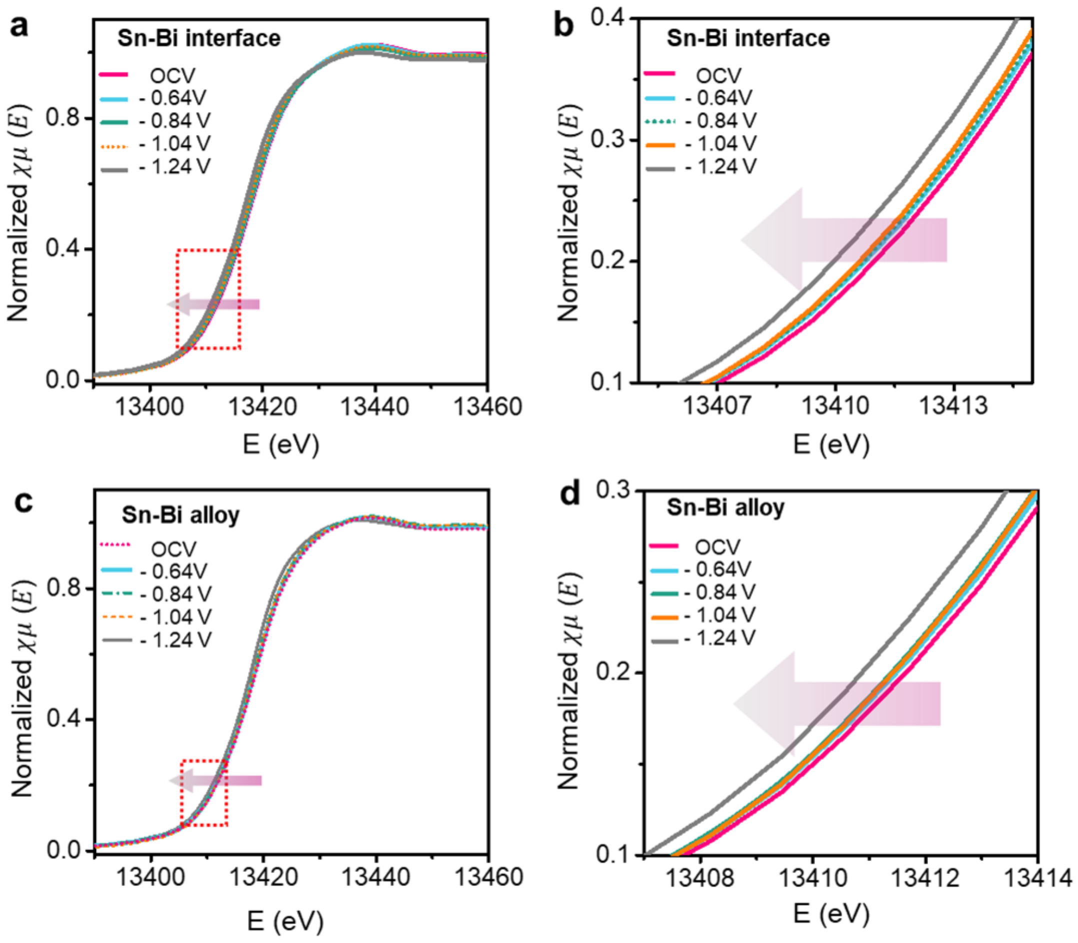

Supplementary Fig. 18. **a, c** *In-situ* XANES spectra of Bi L_3-edge of Sn-Bi interface and alloy samples respectively, at different applied potentials during CO_2RR. **b, d** The enlarged spectra of the red dashed rectangular area in **a, c** respectively**.**

In-situ XANES spectra of Bi L_3-edge of Sn-Bi interface and alloy samples also show the absorption edges slightly shift to the lower energies, indicating a slight reduction of Bi with the cathodic voltages decreasing during CO_2RR. However, the fitting of in-situ EXAFS of Bi L_3-edge presents the fluctuation of the coordination numbers and increased distortion of Bi-Sn and Bi-Bi bonds. This is likely attributed to complex electron density reconfiguration of the system during CO_2RR. And this might be also due to the weakening of signal intensity by the flowing electrolyte.

In-situ X-ray absorption experiments were carried out by using a homemade H-type liquid cell with a φ15 mm Kapton film window (Supplementary Fig. 19). The cell is made of PEEK (poly(ether-ether-ketone)) material. The hard X-ray will pass through Kapton film. Syringe pumps were used to cycle catholyte and anolyte (CO2 saturated 0.5M KHCO3 solution), which were separated by a Nafion 117 membrane to avoid the crossover of produced formate ions. A 10 × 10 mm2 electrode was used as the working electrode, while Pt wire and SCE were counter electrode and reference electrode, respectively. The XANES and EXAFS data of Bi L3-edge (13.42 keV) were collected in BL11B beamline at Shanghai Synchrotron Radiation Facility through employing Si (111) double crystal monochromator and fluorescence mode (Lytle detector). The XANES and EXAFS data of Sn K-edge (29.2 keV) were collected in HXMA beamline at Canadian Light Source, and Si (220) mono crystal was employed. We used fluorescence detector. Tin foil and Bismuth powder standards were collected simultaneously for energy calibration.

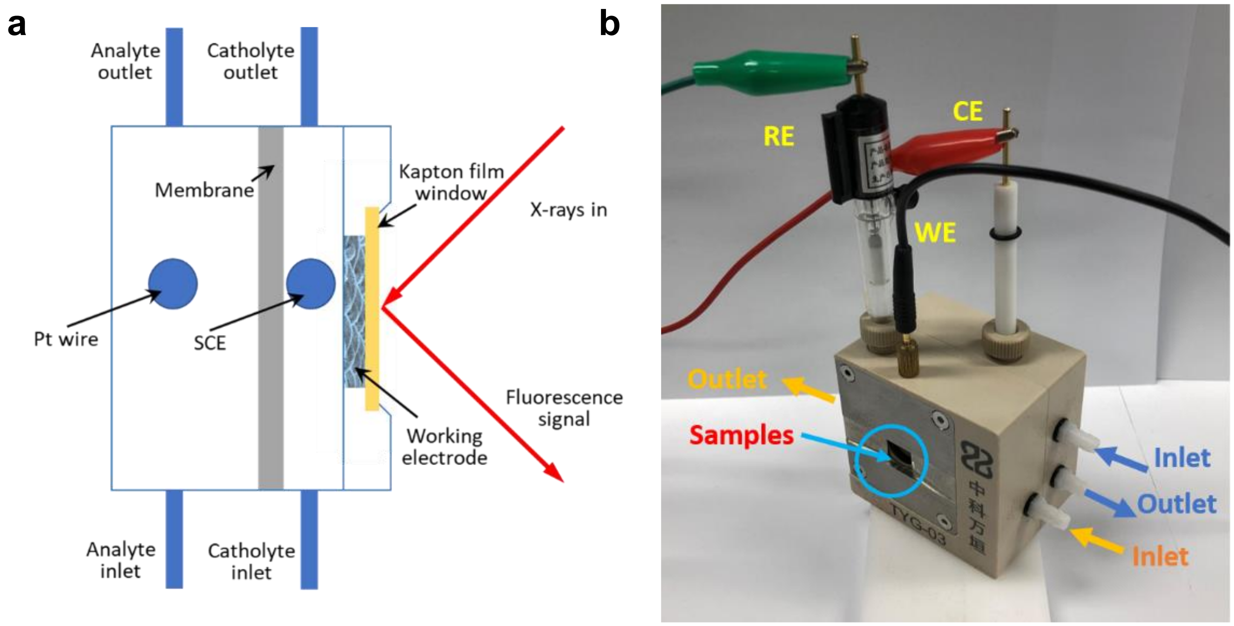

Supplementary Fig. 19. (a) Schematic of the in-situ liquid cell for XAS measurements employed in this study. (b) Photo of the in-situ liquid cell for XAS measurements.

Two samples were measured by *in-situ* XAS: Sn-Bi interface material on carbon fabric and Sn-Bi alloy material on carbon fabric. Two layers of carbon fabric were employed to increase the signal intensity and sticked to the Kapton film window. Both samples have the loading of ~ 2 mg/cm^2 (two layers), and the mass ratios of both Sn and Bi atoms are approximately 20%. Constant potential electrolysis was carried out at different potentials (*i.e.*, OCV, -0.64 V, -0.84 V, -1.04 V, -1.24 V vs. RHE) to monitor the chemical and electronic state changes of the working electrode.

XAS was collected with fluorescence mode when the current density was stable at each potential (after around 20 mins) to guarantee stable chemical and electronic states throughout the electrode and avoid the contingency of the spectra.

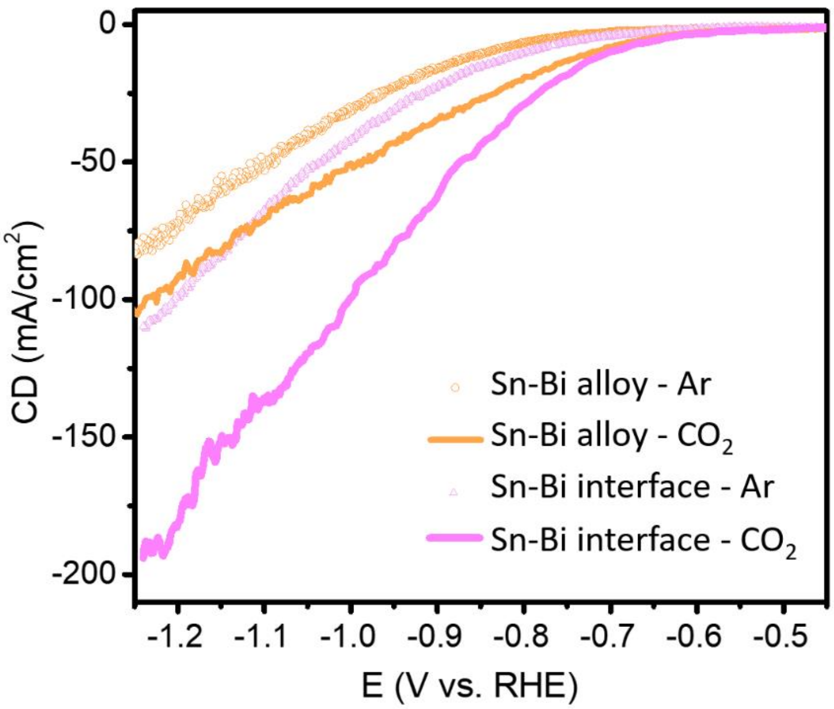

Supplementary Fig. 20 CO_2RR activities (linear sweep voltammetry, LSV curves) in a CO_2-purged (solid line) and Ar-purged (scatters) 0.5 M $KHCO_3$ electrolyte at a scan rate of 20 mV s^{-1} of the prepared Sn-Bi interface and alloy.

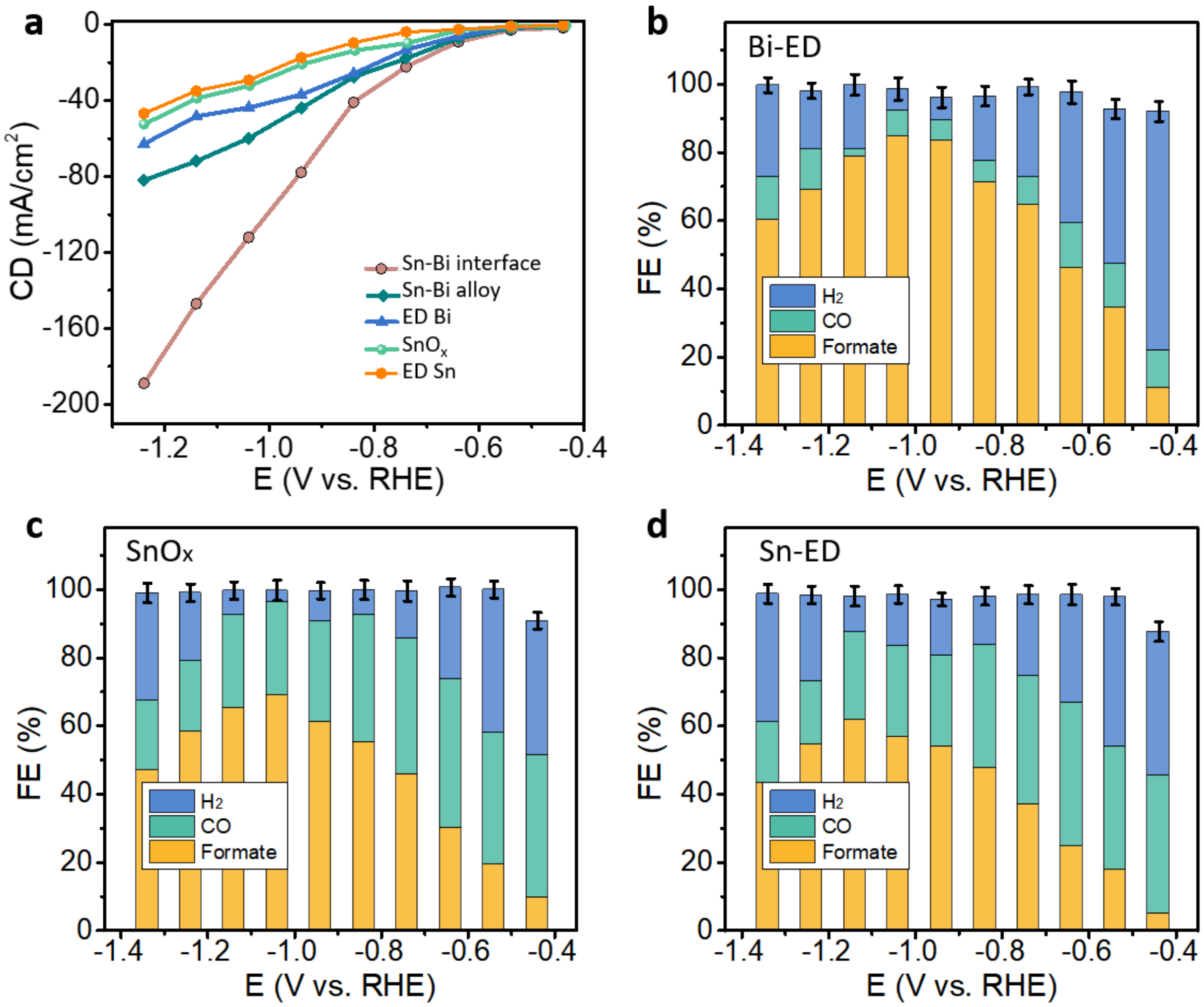

Supplementary Fig. 21 CO_2RR performances. (a) CD for various electrodes. FE toward CO, formate and H_2 for (b) ED-Bi, (c) SnO_x, and (d) ED-Sn.

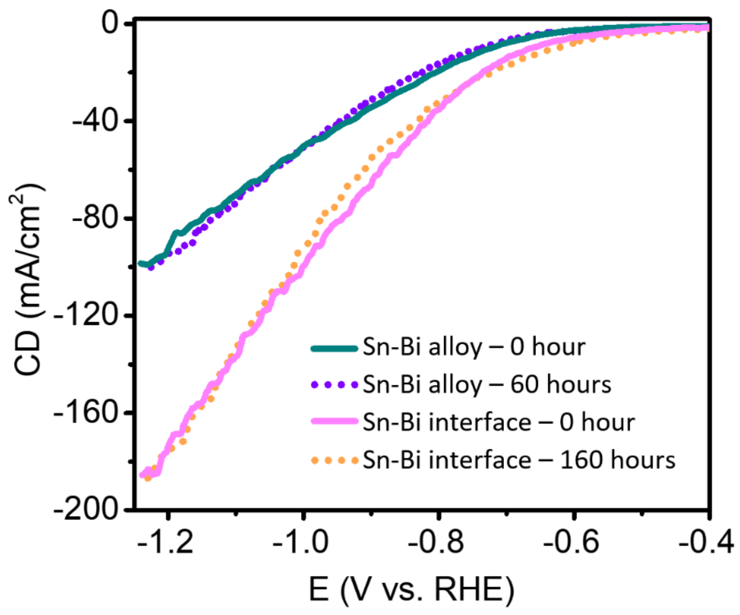

Supplementary Fig. 22 LSV curves of materials before (solid line) and after (scatters) the stability tests.

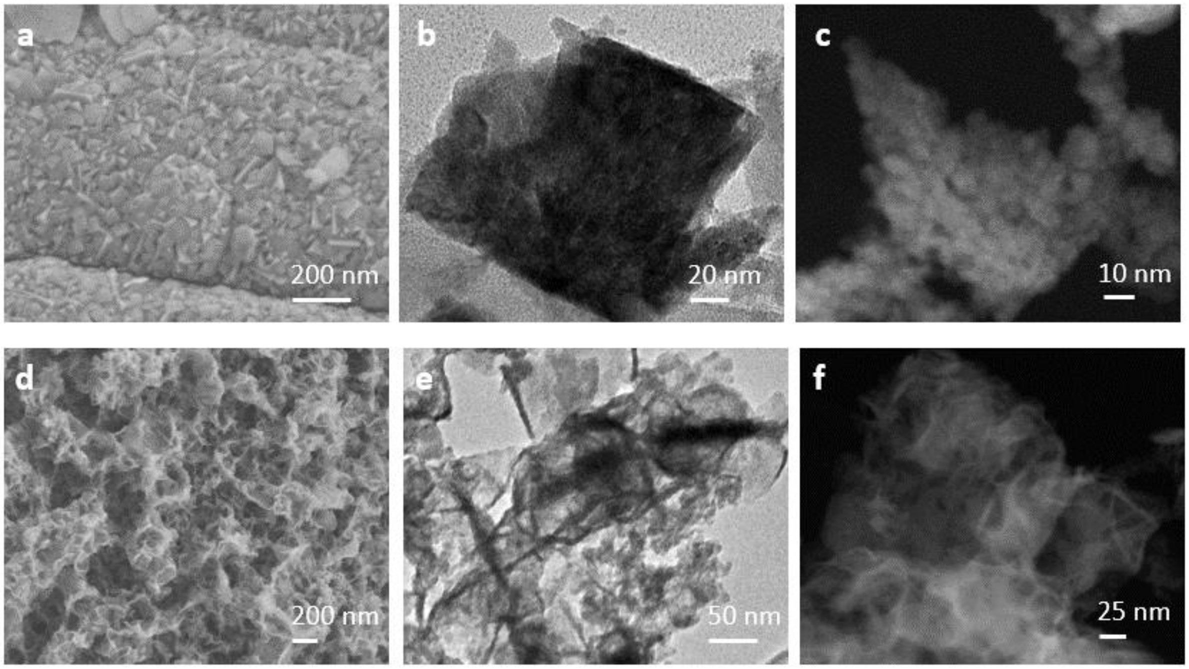

Supplementary Fig. 23 Characterisations of Sn-Bi materials after the stability tests. (a) SEM, (b) TEM, and (c) STEM imagines of Sn-Bi alloy electrode. (d) SEM, (e) TEM, and (f) STEM imagines of Sn-Bi interface electrode.

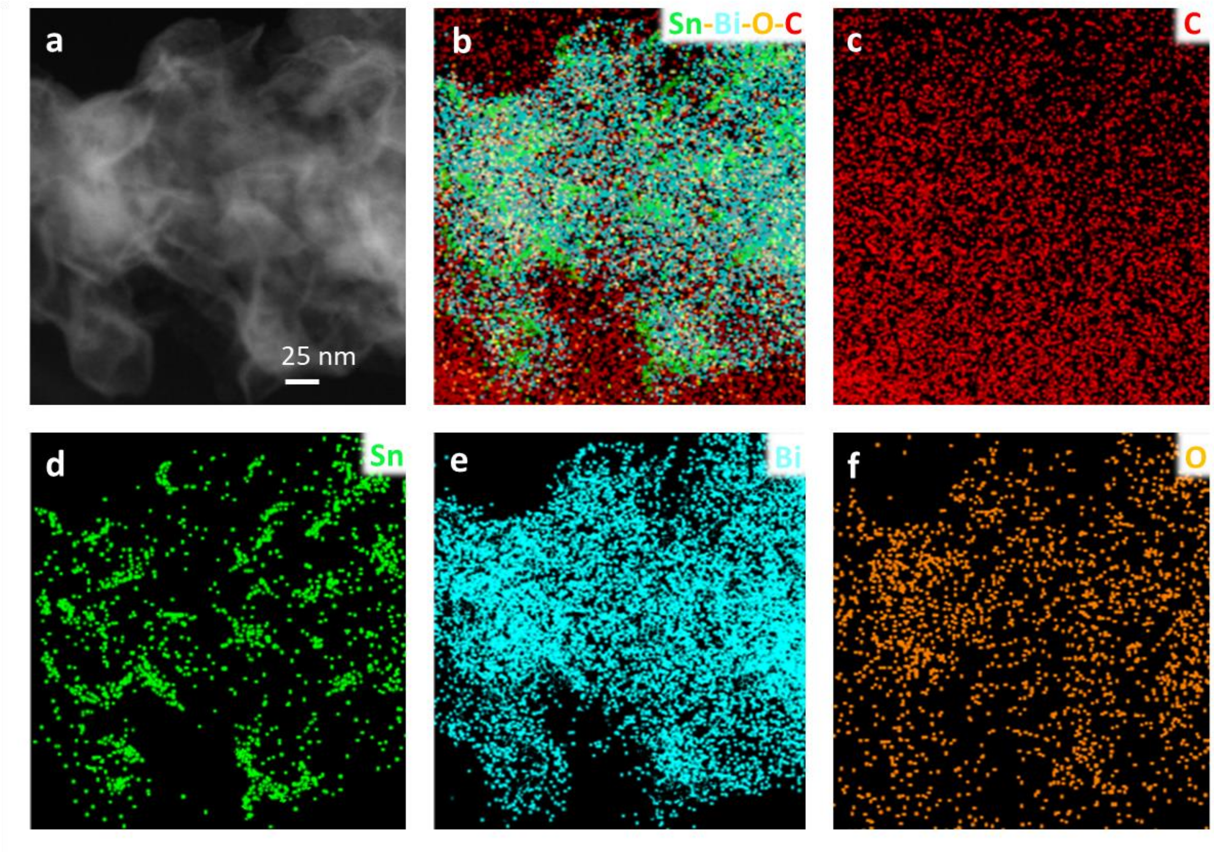

Supplementary Fig. 24 (a) HAADF-STEM image and (b-f) corresponding EDS element mapping of Sn-Bi interface after the stability tests.

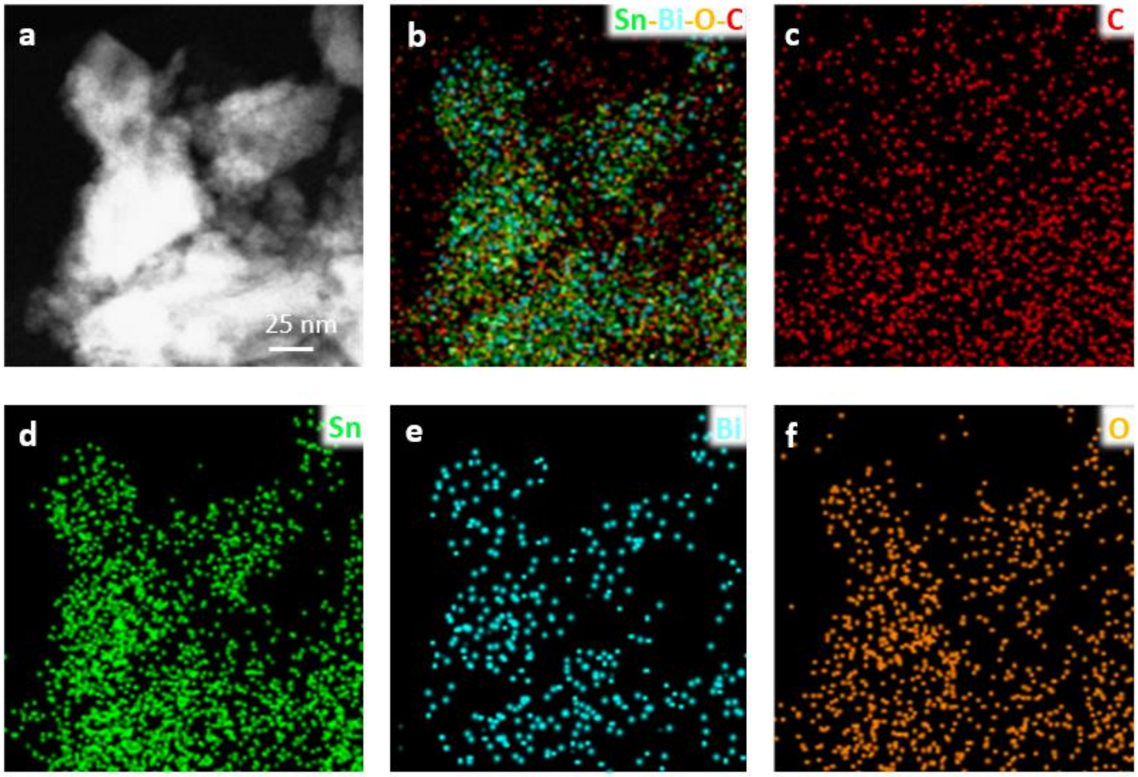

Supplementary Fig. 25 (a) HAADF-STEM image and (b-f) corresponding EDS element mapping of Sn-Bi alloy after the stability tests.

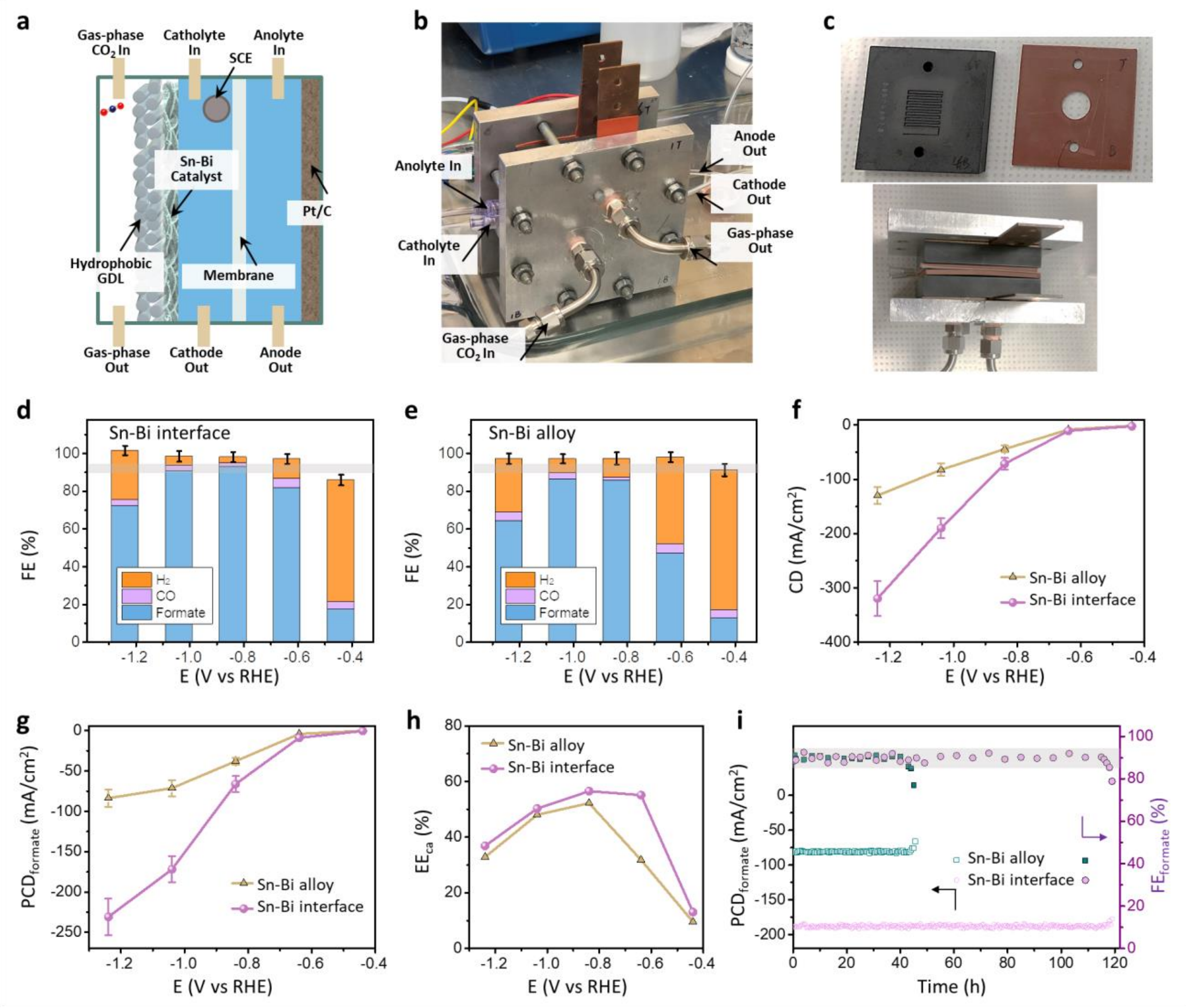

Supplementary Fig. 26. GDE flow cell measurements. (a) Schematic of cell structure. (b) Photographs of homemade GDE flow cell. (c) Photographs of gas diffusion channel plate, electrolyte channel plate, and top view of GDE flow cell. FE of (d) Sn-Bi interface and (e) Sn-Bi alloy catalysts tested in the GDE flow cell under different potentials. (f) CD, (g) $PCD_{formate}$, and (h) cathodic energy efficiency (EE_{ca}) under different potentials. (i) The stability testing of Sn-Bi interface and alloy at -1.04 V vs. RHE.

The CO_2RR was also tested in a homemade flow cell with gas diffusion electrode (GDE). One 1.5 × 1.5 cm^2 Sn-Bi interface catalyst and a hydrophobic gas diffusion layer (GDL) composed the GDE, which was pressed between the gas diffusion channel plate and electrolyte channel plate. The thickness of the electrolyte channel rubber plate was 0.3 cm and the diameter of the electrolyte channel is 1 cm^2. The anode was a commercial Pt/C-CP electrode (Pt/C nanoparticles on carbon paper), and Nafion 117 membrane was applied to avoid crossover of formate ions. The catholyte

(0.5 M $KHCO_3$ solution) was continuously bubbled with CO_2 and circulated by a peristaltic pump. The anolyte was also 0.5 M $KHCO_3$ solution and circulated by a peristaltic pump. The constant potential electrolysis was performed for 30 minutes under various potentials.

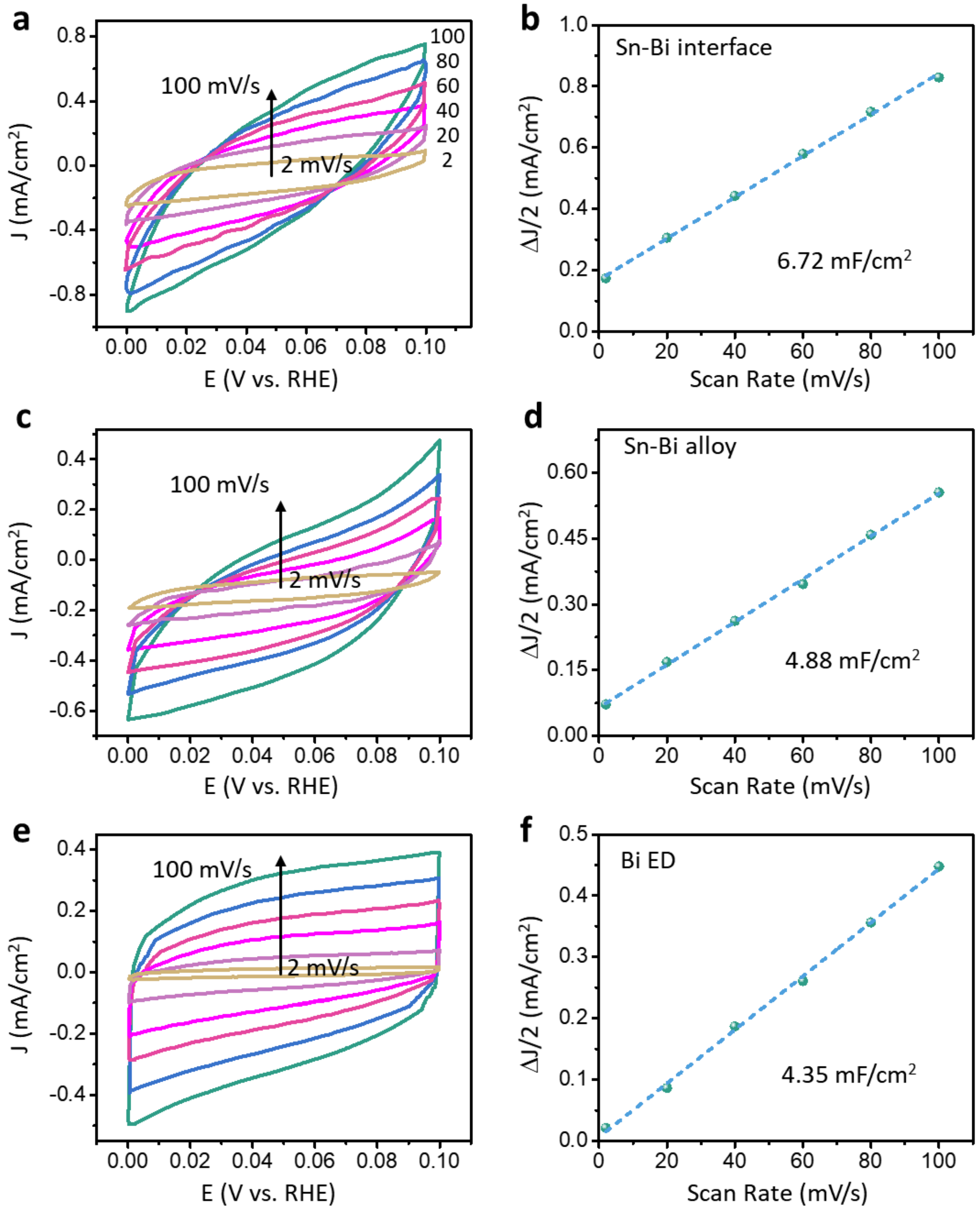

Supplementary Fig. 27. Cyclic voltammograms in Ar-saturated 0.1 M $KHCO_3$ solution with different scan rates in a non-faradaic potential window at different scan rates to determine the Double-layer capacitance (Cdl) over the geometric area. Samples of (a, b) Sn-Bi interface, (c, d) Sn-Bi alloy, (e, f) Bi ED.

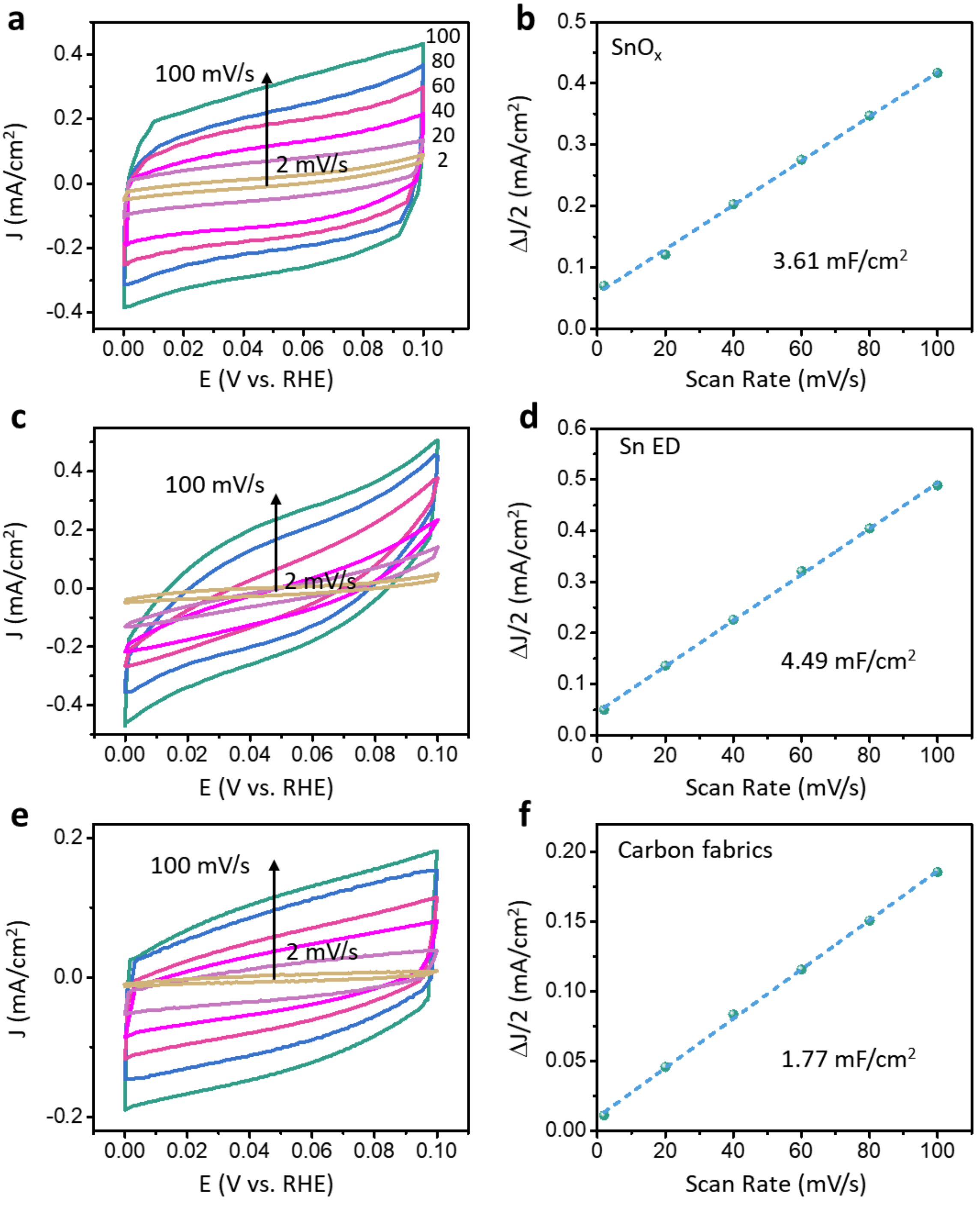

Supplementary Fig. 28. Cyclic voltammograms in Ar-saturated 0.1 M $KHCO_3$ solution with different scan rates in a non-faradaic potential window at different scan rates to determine the Double-layer capacitance (Cdl) over the geometric area. Samples of (a, b) SnO_x, (c, d) Sn ED, (e, f) Carbon fabrics without catalysts.

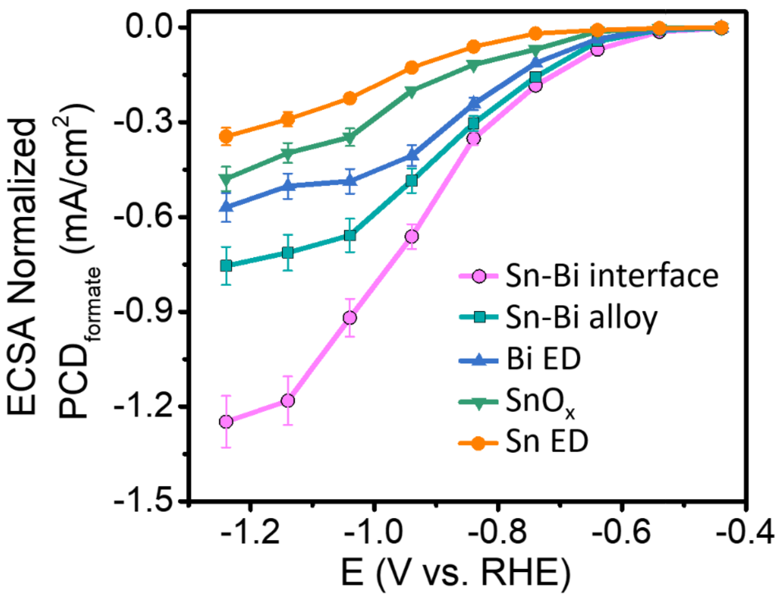

Supplementary Fig. 29. ECSA normalized formate partial current density for various samples: Sn-Bi interface, Sn-Bi alloy, Bi ED, SnO_x, and Sn ED.

3. References

1. T. Ma, Z. Wu, H. Wu, W. Cai, Z. Wen, L. Wang, W. Jin and B. Jia, *Angew. Chem. Int. Ed.*, 2021, **60**, 12554-12559.
2. J. Tang, R. Daiyan, M. B. Ghasemian, S. A. Idrus-Saidi, A. Zavabeti, T. Daeneke, J. Yang, P. Koshy, S. Cheong and R. D. Tilley, *Nat. Commun.*, 2019, **10**, 1-14.
3. G. Wen, D. U. Lee, B. Ren, F. M. Hassan, G. Jiang, Z. P. Cano, J. Gostick, E. Croiset, Z. Bai, L. Yang and Z. Chen, *Adv. Energy Mater.*, 2018, **8**, 1802427.
4. Q. Yang, Q. Wu, Y. Liu, S. Luo, X. Wu, X. Zhao, H. Zou, B. Long, W. Chen, Y. Liao, L. Li, P. K. Shen, L. Duan and Z. Quan, *Adv. Mater.*, 2020, **32**, e2002822.
5. T. Yuan, Z. Hu, Y. Zhao, J. Fang, J. Lv, Q. Zhang, Z. Zhuang, L. Gu and S. Hu, *Nano Lett.*, 2020, **20**, 2916-2922.
6. F. Li, G. H. Gu, C. Choi, P. Kolla, S. Hong, T.-S. Wu, Y.-L. Soo, J. Masa, S. Mukerjee, Y. Jung, J. Qiu and Z. Sun, *Appl. Catal. B*, 2020, **277**, 119241.
7. S. Liu, J. Xiao, X. F. Lu, J. Wang, X. Wang and X. W. Lou, *Angew. Chem. Int. Ed.*, 2019, **58**, 8499-8503.
8. M. Zhao, Y. Gu, W. Gao, P. Cui, H. Tang, X. Wei, H. Zhu, G. Li, S. Yan and X. Zhang, *Appl. Catal. B*, 2020, **266**, 118625.
9. F. Li, L. Chen, G. P. Knowles, D. R. MacFarlane and J. Zhang, *Angew. Chem. Int. Ed.*, 2017, **56**, 505-509.
10. J. Gu, F. Heroguel, J. Luterbacher and X. Hu, *Angew. Chem. Int. Ed.*, 2018, **57**, 2943-2947.
11. Y. Yiliguma, Z. Wang, C. Yang, A. Guan, L. Shang, A. M. Al-Enizi, L. Zhang and G. Zheng, *J. Mater. Chem. A*, 2018, **6**, 20121-20127.
12. C. W. Lee, J. S. Hong, K. D. Yang, K. Jin, J. H. Lee, H.-Y. Ahn, H. Seo, N.-E. Sung and K. T. Nam, *ACS Catal.*, 2018, **8**, 931-937.
13. Z. Chen, K. Mou, X. Wang and L. Liu, *Angew. Chem. Int. Ed.*, 2018, **57**, 12790-12794.
14. F. Lei, W. Liu, Y. Sun, J. Xu, K. Liu, L. Liang, T. Yao, B. Pan, S. Wei and Y. Xie, *Nat. Commun.*, 2016, **7**, 1-8.
15. X. Zhang, Z. Chen, K. Mou, M. Jiao, X. Zhang and L. Liu, *Nanoscale*, 2019, **11**, 18715-18722.
16. P. Deng, H. Wang, R. Qi, J. Zhu, S. Chen, F. Yang, L. Zhou, K. Qi, H. Liu and B. Y. Xia, *ACS Catal.*, 2019, **10**, 743-750.
17. B. Kumar, V. Atla, J. P. Brian, S. Kumari, T. Q. Nguyen, M. Sunkara and J. M. Spurgeon, *Angew. Chem. Int. Ed.*, 2017, **56**, 3645-3649.
18. Q. Zhang, Y. Zhang, J. Mao, J. Liu, Y. Zhou, D. Guay and J. Qiao, *ChemSusChem*, 2019, **12**, 1443-1450.
19. H. Yang, Y. Huang, J. Deng, Y. Wu, N. Han, C. Zha, L. Li and Y. Li, *J. Energy Chem.*, 2019, **37**, 93-96.
20. W. Deng, L. Zhang, L. Li, S. Chen, C. Hu, Z. J. Zhao, T. Wang and J. Gong, *J. Am. Chem. Soc.*, 2019, **141**, 2911-2915.
21. H. Hu, L. Gui, W. Zhou, J. Sun, J. Xu, Q. Wang, B. He and L. Zhao, *Electrochim. Acta*, 2018, **285**, 70-77.
22. W. Luc, C. Collins, S. Wang, H. Xin, K. He, Y. Kang and F. Jiao, *J. Am. Chem. Soc.*, 2017, **139**, 1885-1893.
23. K. Ye, Z. Zhou, J. Shao, L. Lin, D. Gao, N. Ta, R. Si, G. Wang and X. Bao, *Angew. Chem. Int. Ed.*, 2020, **59**, 4814-4821.
24. P. Wang, M. Qiao, Q. Shao, Y. Pi, X. Zhu, Y. Li and X. Huang, *Nat. Commun.*, 2018, **9**.
25. S. Liu, X. F. Lu, J. Xiao, X. Wang and X. W. Lou, *Angew. Chem. Int. Ed.*, 2019, **58**, 13828-13833.
26. K. Fan, Y. Jia, Y. Ji, P. Kuang, B. Zhu, X. Liu and J. Yu, *ACS Catal.*, 2019, **10**, 358-364.
27. N. Han, Y. Wang, H. Yang, J. Deng, J. Wu, Y. Li and Y. Li, *Nat. Commun.*, 2018, **9**, 1-8.
28. M. Zhang, W. Wei, S. Zhou, D.-D. Ma, A. Cao, X. Wu and Q.-L. Zhu, *Energy Environ. Sci.*, 2021.

29. L. Li, A. Ozden, S. Guo, A. d. A. F. P. Garci, C. Wang, M. Zhang, J. Zhang, H. Jiang, W. Wang, H. Dong, D. Sinton, E. H. Sargent and M. Zhong, *Nat. Commun.*, 2021, **12**, 5223.
30. T. Fan, W. Ma, M. Xie, H. Liu, J. Zhang, S. Yang, P. Huang, Y. Dong, Z. Chen and X. Yi, *Cell Reports Physical Science*, 2021, **2**.
31. J. Li, J. Jiao, H. Zhang, P. Zhu, H. Ma, C. Chen, H. Xiao and Q. Lu, *ACS Sustain. Chem. Eng.*, 2020, **8**, 4975-4982.
32. C. Cao, D. D. Ma, J. F. Gu, X. Xie, G. Zeng, X. Li, S. G. Han, Q. L. Zhu, X. T. Wu and Q. Xu, *Angew. Chem. Int. Ed. Engl.*, 2020, **59**, 15014-15020.
33. J. Yang, X. Wang, Y. Qu, X. Wang, H. Huo, Q. Fan, J. Wang, L. M. Yang and Y. Wu, *Adv. Energy Mater.*, 2020, **10**, 2001709.
34. C. Xia, P. Zhu, Q. Jiang, Y. Pan, W. Liang, E. Stavitski, H. N. Alshareef and H. Wang, *Nat. Energy*, 2019, **4**, 776-785.
35. L. Fan, C. Xia, P. Zhu, Y. Lu and H. Wang, *Nat. Commun.*, 2020, **11**, 3633.
36. K. Ye, Z. Zhou, J. Shao, L. Lin, D. Gao, N. Ta, R. Si, G. Wang and X. Bao, *Angew. Chem. Int. Ed. Engl.*, 2020, **59**, 4814-4821.
37. A. Del Castillo, M. Alvarez-Guerra, J. Solla-Gullón, A. Sáez, V. Montiel and A. Irabien, *Journal of CO2 Utilization*, 2017, **18**, 222-228.
38. C. Liang, B. Kim, S. Yang, Y. Liu, C. F. Woellner, Z. Li, R. Vajtai, W. Yang, J. Wu and P. J. Kenis, *J. Mater. Chem. A*, 2018, **6**, 10313-10319.
39. S. Verma, Y. Hamasaki, C. Kim, W. Huang, S. Lu, H.-R. M. Jhong, A. A. Gewirth, T. Fujigaya, N. Nakashima and P. J. A. Kenis, *ACS Energy Lett.*, 2018, **3**, 193-198.
40. C. M. Gabardo, A. Seifitokaldani, J. P. Edwards, C.-T. Dinh, T. Burdyny, M. G. Kibria, C. P. O'Brien, E. H. Sargent and D. Sinton, *Energy Environ. Sci.*, 2018, **11**, 2531-2539.
41. F. P. Garcia de Arquer, C. T. Dinh, A. Ozden, J. Wicks, C. McCallum, A. R. Kirmani, D. H. Nam, C. Gabardo, A. Seifitokaldani, X. Wang, Y. C. Li, F. Li, J. Edwards, L. J. Richter, S. J. Thorpe, D. Sinton and E. H. Sargent, *Science*, 2020, **367**, 661-666.
42. C. T. Dinh, T. Burdyny, M. G. Kibria, A. Seifitokaldani, C. M. Gabardo, F. P. Garcia de Arquer, A. Kiani, J. P. Edwards, P. De Luna, O. S. Bushuyev, C. Zou, R. Quintero-Bermudez, Y. Pang, D. Sinton and E. H. Sargent, *Science*, 2018, **360**, 783-787.
43. C. P. O'Brien, R. K. Miao, S. Liu, Y. Xu, G. Lee, A. Robb, J. E. Huang, K. Xie, K. Bertens, C. M. Gabardo, J. P. Edwards, C.-T. Dinh, E. H. Sargent and D. Sinton, *ACS Energy Lett.*, 2021, **6**, 2952-2959.
44. Y. Xu, J. P. Edwards, S. Liu, R. K. Miao, J. E. Huang, C. M. Gabardo, C. P. O'Brien, J. Li, E. H. Sargent and D. Sinton, *ACS Energy Lett*, 2021, **6**, 809-815.
45. J. A. Rabinowitz and M. W. Kanan, *Nat. Commun.*, 2020, **11**, 5231.
46. F. Pan and Y. Yang, *Energy Environ. Sci.*, 2020, **13**, 2275-2309.
47. C. Tang, W. Wang, A. Sun, C. Qi, D. Zhang, Z. Wu and D. Wang, *ACS Catal.*, 2015, **5**, 6956-6963.
48. M. W. Gu, H. H. Peng, I. P. Chen and C. H. Chen, *Nat. Mater.*, 2021, **20**, 658-664.
49. J. K. Nørskov, F. Abild-Pedersen, F. Studt and T. Bligaard, *Proc. Natl. Acad. Sci. U.S.A.*, 2011, **108**, 937-943.
50. B. Hammer and J. K. Nørskov, *Adv. Catal.*, 2000, **45**, 71-129.
51. H. Liu, J. Xia, N. Zhang, H. Cheng, W. Bi, X. Zu, W. Chu, H. Wu, C. Wu and Y. Xie, *Nat. Catal.*, 2021, **4**, 202-211.
